# Generalizable deep learning framework for 3D medical image segmentation using limited training data

**DOI:** 10.1186/s41205-025-00254-1

**Published:** 2025-03-06

**Authors:** Tobias Ekman, Arthur Barakat, Einar Heiberg

**Affiliations:** 1 Department of Medical Imaging and Physiology, Lund University, Lund, Sweden; 2 Department of Medical Imaging and Physiology, Skåne University Hospital, Lund, Sweden; 3 Wallenberg Center for Molecular Medicine, Lund University, Lund, Sweden

**Keywords:** Segmentation, Machine learning, Artificial intelligence, Deep learning, 3D printing

## Abstract

Medical image segmentation is a critical component in a wide range of clinical applications, enabling the identification and delineation of anatomical structures. This study focuses on segmentation of anatomical structures for 3D printing, virtual surgery planning, and advanced visualization such as virtual or augmented reality. Manual segmentation methods are labor-intensive and can be subjective, leading to inter-observer variability. Machine learning algorithms, particularly deep learning models, have gained traction for automating the process and are now considered state-of-the-art. However, deep-learning methods typically demand large datasets for fine-tuning and powerful graphics cards, limiting their applicability in resource-constrained settings. In this paper we introduce a robust deep learning framework for 3D medical segmentation that achieves high performance across a range of medical segmentation tasks, even when trained on a small number of subjects. This approach overcomes the need for extensive data and heavy GPU resources, facilitating adoption within healthcare systems. The potential is exemplified through six different clinical applications involving orthopedics, orbital segmentation, mandible CT, cardiac CT, fetal MRI and lung CT. Notably, a small set of hyper-parameters and augmentation settings produced segmentations with an average Dice score of 92% (SD = ±0.06) across a diverse range of organs and tissues.

**Supplementary Information:**

The online version contains supplementary material available at 10.1186/s41205-025-00254-1.

## Introduction

Medical image segmentation is essential for numerous clinical applications, such as quantification and visualisation, which enable improved diagnostics and advancements in medical research. This study focuses on segmentation of anatomical structures for 3D printing and advanced visualization such as virtual or augmented reality [1].

Manual or semi-automated segmentation is a tedious, resource intensive task that leaves room for user error and variability [2]. Therefore, there has been considerable interest in the development of automated segmentation algorithms over the past decade [3]. The main focus of the automation is to decrease manual work required to do high quality segmentation. Ultimately, the user is responsible to ensure accuracy and make manual adjustments if necessary.

During the last decade, deep convolutional neural networks (CNNs) have consistently surpassed hand-crafted segmentation algorithms in performance [4] . One of the most widely used networks is the 2D U-Net architecture [5]. The 2D U-Net architecture is capable of segmenting 3D medical images by segmenting the volume slice by slice. Consensus techniques, which are well-established, comprise independently segmenting each voxel along three orthogonal directions-axial, sagittal, and coronal-and subsequently combining these predictions to determine the final voxel value [6, 7]. Further, the U-Net architectures have been expanded to a 3D version [8], allowing for full volumetric segmentation, and to the generalised nnU-Net framework [9]. The latter automatically configures a U-Net network and adjusts hyperparameters based on the training data. The nnU-Net framework has excelled in a multitude of segmentation challenges [9].

Despite notable advancements and high performing algorithms, many automatic-segmentation algorithms have not been clinically implemented due to various factors [10]:

- CNN-based segmentation approaches typically require large training datasets to achieve accuracy levels that offer clinical benefits, meaning that the combination of automated segmentation and manual corrections result in significant time savings compared to using manual segmentation alone.
- Automated segmentation algorithms need to be combined with efficient methods for manual segmentation to allow the user to correct potential errors.
- The algorithms need to be incorporated into systems that fits with the clinical routine and interface with hospital PACS systems.
- Stringent regulatory requirements mandate both validation and adherence to a certified quality management system throughout the development process.
- Many hospitals lack powerful compute servers, creating a need for algorithms capable of running on standard consumer-grade gaming graphics cards. An alternative approach is to utilize cloud-based solutions, however such strategies comes with complexity in terms of cyber-security and data integrity concerns.

To address several of these clinical translation challenges, we propose a generic framework for 3D medical image segmentation where the aim is to streamline development of CNN-based segmentation algorithms for different clinical applications and minimize the time and resources needed for clinical deployment. Key features of the framework include:

- Data efficiency and high performance using limited training data.
- Pre-configured settings requiring little or no hyper-parameter tuning for optimal performance.
- Computational efficiency, allowing the inference to be run on standard consumer-grade graphics cards.
- Easy adaptation of regulatory submissions for new applications by using the framework both for training and inference procedures.
- The difference between clinical applications is only driven by different training and testing data.

To demonstrate the framework’s general applicability to different segmentation tasks, we trained and evaluated its performance across six distinct segmentation tasks, each presenting specific challenges, modality and tissue types. The tasks are all within the field of 3D printing but the methods are applicable to other fields such as visualization, or quantification. Further details on the cohorts and the specific clinical applications are provided in the Cohorts section.

## Methods

### Network architecture

**Fig. 1:**
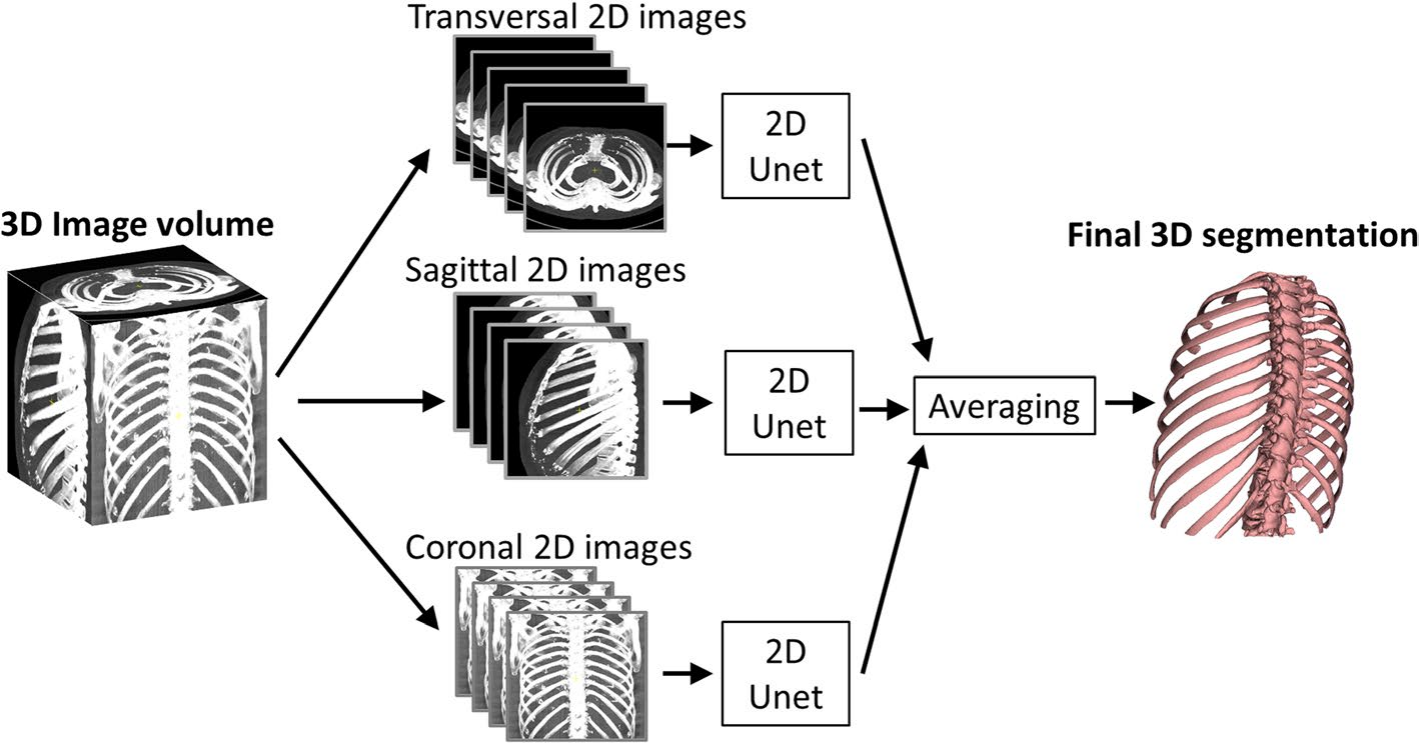
Inference workflow. The 3D image volume is split into three stacks of 2D images in transversal, sagittal, and coronal directions, respectively. The same 2D U-Net CNN performs inference on all three stacks of 2D images. This results in three image volumes. The final voxel-wise classification is achieved by averaging the probabilities

The used underlying network is the standard 2D U-Net, which was chosen as it is the most widely used CNN for medical image segmentation [5]. An overview of the inference workflow is shown in Fig. 1. Our approach uses Mathworks implementation of the network, which adheres to the core network structure but allow for certain configurable aspects. Our network employs an encoder, with 3x3 convolutional filters and ’same’ padding, preserving the input dimensions throughout the network, consistent with the original design. While the foundational architecture is unchanged, one difference in our approach is that we segment images in smaller patches rather than full volumes, which optimizes memory usage without affecting the accuracy of segmentation. Thus, while our model remains largely consistent with the U-Net proposed by Ronneberger et al., these minor modifications-such as patch-based segmentation and flexible hyperparameters-are introduced to enhance computational efficiency and adaptability to various input sizes.

Initially, the image volume is resampled to isotropic image resolution determined by the highest resolution in-plane or through plane of the original DICOM data. The image volume is thereafter divided into three stacks of 2D images in the transversal, sagittal and coronal direction. The segmentation is performed with a **single** 2D U-Net applied to the separate stacks of 2D images. As the U-Net has a fixed input and output image size, each slice is subdivided into patches of uniform size. The final result is achieved by averaging the probabilities from each stack of 2D patched slices. This strategy enables a lightweight and efficient 2D U-Net to perform a 3D classification task. By using three orthogonal directions, 3D information is implicitly incorporated into the model. The framework was implemented in Matlab R2022a (Mathworks, USA) and was incorporated into the clinically available software Segment 3DPrint (Medviso AB, Lund, Sweden).

### Training and data augmentation

In the training process, a stack of images from either CT or MR scans were fed to the network and weights and biases were adjusted by optimizing a cross entropy loss function using the standard Adam optimizer. The image volumes were then divided into multiple 128x128 pixel patches with specified overlaps to ensure comprehensive coverage. The patches were extracted from slices intersecting the same 3D positions, ensuring that each region is segmented multiple times. The overlap between patches guarantees that boundary areas are not missed. In order to improve network performance, enhance generalization, and reduce the amount of training data, augmentation was used when extracting the patches. All applications used the same augmentation with minimal modifications^1^. Augmentations were primarily applied to individual patches, except for global rotation augmentation, which rotates the entire initial volume before patch generation. The complete list of augmentations is provided in Table 1. It is worth noting that no mirroring or transposing operations were applied when training the mandible network. This decision was deliberate, as for this application the positioning and orientation of the mandible relative to the rest of the skull is crucial information for the network to effectively distinguish between them. Indeed, both classes consist of the same tissue types, resulting in similar intensity levels on CT images and in the corresponding patches. In contrast, for other multiclass networks such as the lung and trachea network, each class comprises different tissue types with distinct attenuation properties, leading to varying intensity levels on CT images and patches. This variation facilitates classification, even in the absence of positional features. Settings for scaling and rotation were configured according to the potential variation in scaling and rotation observed across different patients’ images. No tuning was performed after setting parameters in consensus. Augmentation was performed differently for each epoch to avoid that the networked encounter the exact same patch more than once.

**Table 1:** Training augmentations

Augmentation	Probability	Distribution	Unit
3D Rotation^a^	0–50 %	U(−20, 20)	Degrees
2D Rotation	20–25 %	U(−20, 20) - U(−45, 45)	Degrees
Scaling	20–100 %	U(0.7, 1.3) - U(0.6, 1.4)	Factor
Mirroring H.^b^	50 %^d,e^	-	-
Mirroring V.^c^	50 %^d,e^	-	-
Transpose	50 %^d,e^	-	-
White noise	10–15 %	U(0, 15 %)	Of intensity range
Streak noise	10–20 %^e^	U(0, 50 %) - U(0, 70%)	Of intensity range
Brightness	15–25 %	U(−30, 30) - U(−100, 100)	HU
Contrast	15–25 %	U(0.95, 1.05) - U(0.7, 1.3) - U(0.65, 1.5)	Factor
Gaussian blur	25 %	{3}	Filter size (pixels)
Sharpening	15 %	U(0.7, 1.3)	Only fetal

U denotes uniform random distribution within the given range

^a^Global Rotation applied to the initial volume

^b^Mirroring Horizontally

^c^Mirroring Vertically

^d^For the mandible network : 0 %

^e^For the fetal network : 0 %

### Hyper-parameters

Fine-tuning hyper-parameters is a crucial step in obtaining optimal segmentation results within any machine learning framework [11]. In our current framework, emphasis was placed on enhancing generalizability to facilitate adoption across diverse user domains. Consequently, our aim was to identify hyper-parameters that could remain consistent across various application areas (Table 2).

**Table 2:** Hyper-parameters for Model Training

Hyper-parameter	Value
Patch size	128^a^
Learning rate	0.001^a^
Learning rate drop period	4 - 6
Learning rate drop factor	0.8 - 0.9
Encoder depth	4
Optimization method	Adam
L2 regularization strength	1e-4
Gradient Threshold Method	L2 norm
Gradient decay factor	0.9
Squared gradient decay factor	0.9990
Epochs	100
Minimum batch size	30 - 100
Training / testing type	Hold-out ^a^

^a^For fetal network

Patch size : 256

Learning rate : 0.005

Training/ testing type : 5-fold cross-validation

### Cohorts

Six cohorts were considered in this paper focusing on different segmentation challenges especially prominent in the field of 3D printing [12]:

- Skeletal structures
- Oculo-cranial structures
- Mandibulo-cranial structures
- Congenital heart defects
- Fetal anatomy
- Pulmonary-tracheal structures

An overview of the six cohorts is shown in Table 3. Most cases were drawn from individuals undergoing clinical procedures referred for generation of anatomical model to the 3D Centre of Skåne University Hospital. When collecting the cohorts we ensured to capture extensive diversity across anatomy, pathology, age, image resolutions, scanners, and scanning parameters. This diversity was preserved when dividing the images into training and test datasets. All subjects were completely anonymized and the retained information only comprised approximate age at the time of scanning, sex, scanning parameters, and minimal details regarding anatomical region or primary pathology. The Swedish Ethical Review Authority waived informed consent (Dnr 2021–03583) for these subjects. For each of the cohorts there is a supplemental file with detailed information on age, sex, pathology, image resolutions, and size of image volume. All image data was resampled to isotropic resolution. The used image resolution was the highest of either in-plane or through plane resolution of the original DICOM data.

**Table 3:** Cohort information

Anatomy	Modality	Train size	Test size	Application and anatomical region
Skeletal structures	CT	3–40	13	Pre-surgical planning, creation of drilling and cutting guides. Data included anatomical parts of the body such as head, shoulders, extremities, spine, pelvis, hands including radius and ulna, and feet. Knees were exclusively included in the test dataset. Segmented object was bone structures where the bones were completely filled.
Oculo-cranial structures	CT	21	10	Creation of anatomical models for pre-bending orbital floor implants, design of orbital floor implants and neurosurgical skull implants. Included object was bones including the orbital floor.
Mandibulo-cranial structures	CT	19	10	Pre-surgical planning for mandibular reconstruction.
Congenital heart defects	CT	32	10	Surgery planning before either surgical or catheter based intervention of congenital heart defect. Segmented structures were bones and intra-cardiac and cardiovascular blood pool.
Fetal anatomy	MR	20	22	Improving prenatal diagnosis, the capability to estimate fetal weight and use this to normalize blood flow measurements.
Pulmonary-tracheal Structures	CT	15	10	Pre-surgical planning of cardiac surgery or catheter interventions where it is often important to model the location of the trachea in conjunction to cardiovascular structures.

Modality refers to imaging modality and train size refers to the number of patients used for training, and test size the number of patients used for testing

#### Skeletal structures

The clinical application of this cohort is general bone segmentation. Typical 3D printing use cases are musculoskeletal applications such as anatomical models; virtual planning of osteotomy where cutting and drilling guides are designed. Other potential applications could be bone tumors or to locate other defects in relation to skeletal structures. The anatomical areas covered by the different cases is essentially the entire body, such as hand and shoulder, feet, ankle, spine, ribs and skull. The cohort includes a large range underlying pathology such as severe fractures, tumors, malunions, and scoliosis. In addition to subjects from Skåne University Hospital, 8 cases were included from The Cancer Imaging Archive (TCIA) [13] in order to include older scans.

**Fig. 2:**
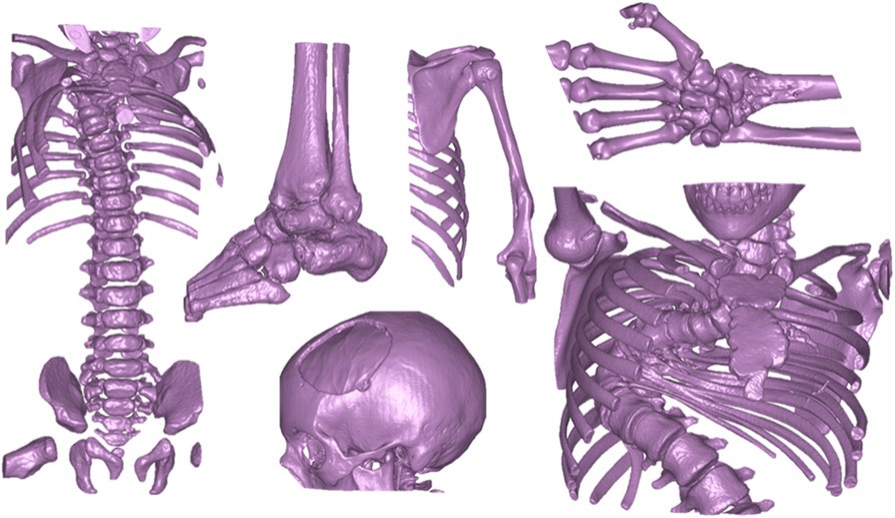
Skeletal structure ground truth segmentations

The specific challenge with this segmentation task is the large variability in the anatomy. Another challenge is that the ground truth bones are filled to not only include the cortical bone, but also the spongeous bone. For this segmentation task only one object class was used; *bone*. Typical examples of delineations are shown in Fig. 2.

#### Oculo-cranial structures

The clinical application of the orbital cohorts is segmentation of the thin orbital floor structure. During blunt skull trauma the orbital floor that holds the eyes may fracture resulting in the eye dropping down causing blurred vision and entrapped ocular muscles. Typically, an orbital floor fracture is fixed with either a bent thin titanium plate or a patient-specific orbital floor plate. In surgical preparation, a commonly employed technique involves segmenting the healthy orbit, when feasible, and mirroring it for the purpose of pre-bending a plate or manufacturing a patient-specific plate. However, manually segmenting the orbital floor is challenging due to its thin structure, which is often barely discernible on CT scans. Furthermore, we also included the eyes in the segmentation task as this allows rapid detection if the blunt trauma have caused one of they eyes to drop. The underlying pathologies in the cohorts included skull trauma, orbital floor fractures, cranioplasty, and suspected stroke. For this segmentation task, two object classes were used; *bone* including the thin orbital floor, and *eyes*.

**Fig. 3:**
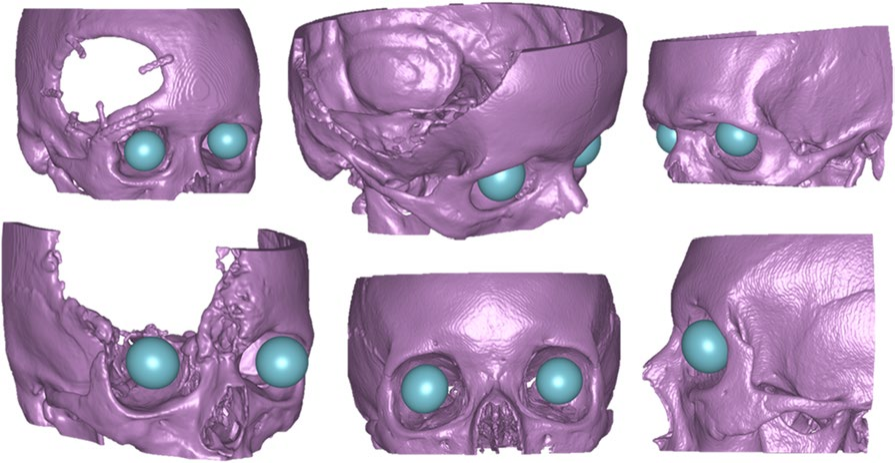
Oculo-cranial structure ground truth segmentations

The specific challenge with this segmentation task is to properly segment the thin orbital floor. Typical examples of delineations are shown in Fig. 3.

#### Mandibulo-cranial structures

**Fig. 4:**
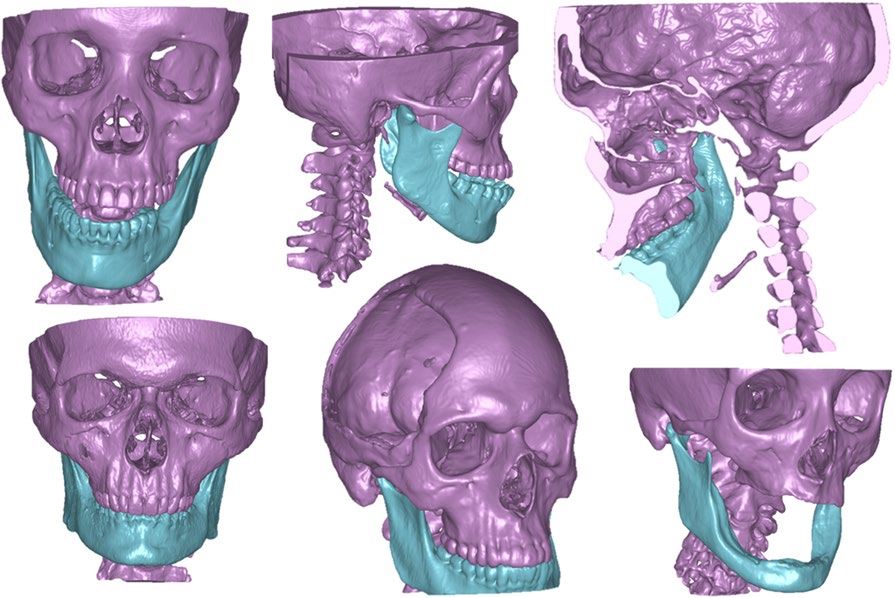
Mandibulo-cranial structure ground truth segmentations

Separation of the mandible and the maxilla is often required in surgical planning of maxillofacial cases. This splitting is often time consuming to perform manually, especially if a distance plate is not used to separate teeth, or when numerous artifacts are present due to dental fillings or procedures. The underlying pathology in the study included skull trauma, orbital floor fracture, cranioplasty, orthogonathic surgery and tumors. For the segmentation task two classes were used; *mandible* and *cranium* in which the latter included all bones except the mandible. Typical examples of delineations are shown in Fig. 4.

The specific challenge with this segmentation task is the separation between maxillary teeth from mandibular teeth. This task requires that the network learns the shape and position of the mandible relative to the maxilla. Hence, during both training and inference only two orientations sagittal and coronal were used. This is because distinguishing the mandible from the rest of the cranium becomes nearly impossible when analyzing 2D patches taken in the transversal plane.

#### Congenital heart defects

The data consisted of a wide range of congenital heart defect cases with an age distribution ranging from 1 day to 18 years old. The pathologies included a wide range of congenital heart defects such as tetralogy of Fallot, univentricles, major pulmonary artery connection, hypoplastic ventricles, double outflow ventricles and several types of ventricular septum defects. All images were acquired with intravenous contrast injected. For this segmentation task two classes were used; *blood pool* encompassing the chambers of the heart and connected blood vessels with contrast and *bone*.

**Fig. 5:**
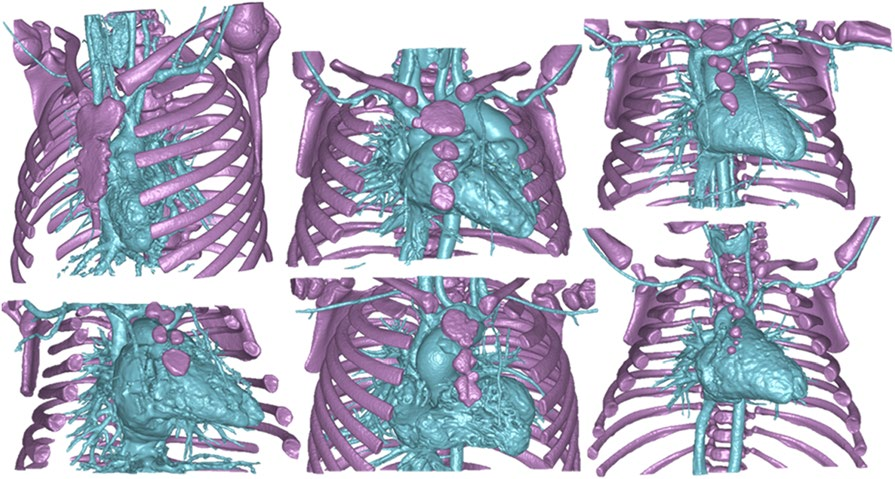
Congenital heart defect ground truth segmentations

The specific challenge with this segmentation task is the large anatomical variations between patients. Additionally, the utilization of contrast introduced significant differences in intensity levels for similar tissues. Indeed, the contrast distribution varies considerably based on multiple factors related to the patient, the contrast medium, how it is injected, timing between injection and imaging, and the CT scanner characteristics [3]. Typical examples of delineations are shown in Fig. 5.

#### Fetal anatomy

**Fig. 6:**
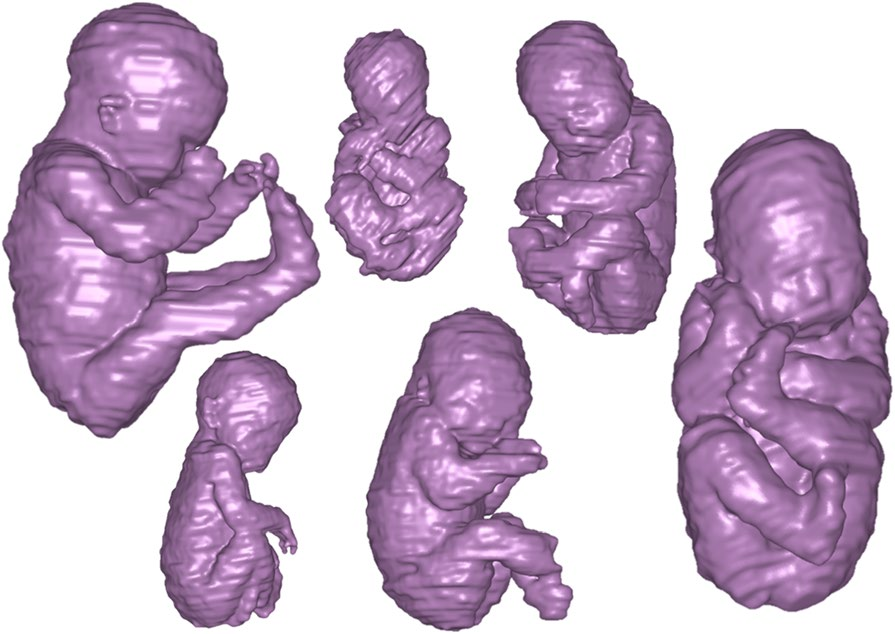
Fetal anatomy ground truth segmentations

The fetal application was included to show that the proposed framework also work for MR images. The fetal cohort is the same cohort as presented by Ryd et al. [14]. In summary, forty-two fetuses (gestational age 36 (29–39) weeks) were included. Fetal MRI examinations were performed both on clinical indication and for research aimed at developing fetal cardiovascular MRI. The cohort consisted of fetuses with and without known or suspected congenital heart disease. For this segmentation task four classes were used; *fetus*, *placenta*, *umbilical cord* and *amniotic fluid*. The primary focus in the study was the fetus, however other intrauterine structures were added in order to enhance network performance by providing additional information. Only the segmentation of the fetus will be investigated in this paper. The regional ethics committee approved the study (Dnr 2013/551). All pregnant women gave written informed consent before participation in the study. Typical examples of delineations are shown in Fig. 6.

#### Pulmonary-tracheal structures

The clinical use case for this segmentation task is visualisation and 3D printing of pulmonary disease pathologies as well as for cardiovascular applications where it often is of high importance to see the location of the trachea in relation to the great arteries. Specific examples in congenital heart defects are aortic rings or instances where vessels impede airflow in the trachea. The training data consisted of 15 patients with a wide range of clinical conditions. Examples of the clinical conditions included are intracranial aneurysm, lung cancers, esophagus atrophy, brachialis compression on trachea, aortic coarctation, aortic ring, and several congenital heart defect cases. 11 out of 15 data sets had contrast on board. The test cases comprised 10 patients and the pathologies included a tumour on the trachea, two lung cancer data sets, five congenital heart defects, including one case with aortic ring, and two adolescents with congenital heart defects where one had a pacemaker. Out of the 10 test sets 7 had contrast onboard. For this segmentation task three classes were used; *lung tissue*, *lung vessels* (blood vessels inside the lungs), and *trachea*. Testing was performed on *lung tissue* and *trachea*, separately.

**Fig. 7:**
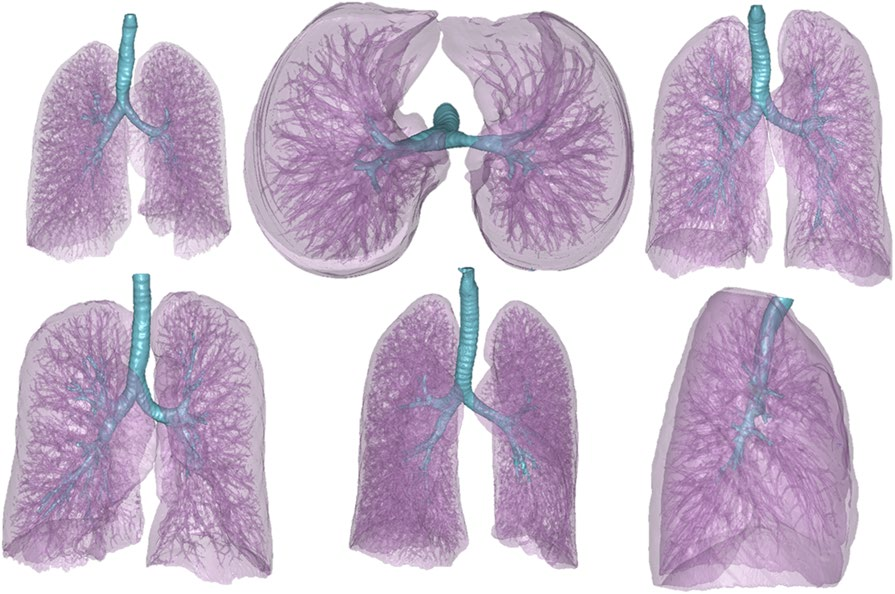
Pulmonary-tracheal structure ground truth segmentations

The specific segmentation challenges for this cohort were the separation of the lungs and the distal branches of the trachea. The inclusion of this segmentation task also serves a second purpose; to evaluate the behaviour of the proposed framework to perform under severe class imbalance, where the trachea was only 1.8%±0.7% of the lung volume. The third challenge with the cohort is that for some data sets there was contrast injected and for other datasets no contrast was used. Typical examples of delineations are shown in Fig. 7.

### Image acquisitions

Imaging was performed on all of the following CT vendors, Siemens, Philips, General Electric (GE), and Toshiba/Canon. The used scanner and image resolution for each case is given in the supplemental material. All fetal MRIs were performed using a balanced steady-state free precession sequence on a 1.5 T Aera scanner by Siemens Healthineers in Erlangen, Germany.

### Ground truth labeling

Manual segmentations of the tissues were performed using Segment 3DPrint v4.0 (Medviso AB, Lund, Sweden). The manual segmentation consisted of a semi-automatic approach such as thresholding and morphological operations. Subsequently, extensive manual corrections and adjustments were performed slice by slice and by using a 3D pen tool. All data sets were reviewed by a second observer and the delineations were used as both ground truth for training of neural networks as well as for evaluation of network performance. There are no overlap of data between test and training data.

### Post processing

For all applications, the objects were filled and objects smaller than 0.1ml were removed. The framework includes a possibility to set three configurable processing parameters, see Table 4.

**Table 4:** Post-processing settings for various structures

Name	Probability threshold	n-largest object	Touch other objects
Skeletal	50%	-	No
Orbita	12.5%	1	No
Eyes	12.5%	2	No
Mandible	50%	1	No
Cranium	50%	1	No
Congenital	25%	1	No
Fetal	50%	1	No
Parenchyma	50%	2	No
Trachea	50%	1	Yes, Parenchyma

First column show the required probability to classify a voxel to belong to the class. Second column show at most how many objects of the class that is expected. Third column determines if objects of a class is required to touch objects of another class

#### Increased probability threshold

The network outputs for each class including the background a likelihood of belonging to a specific class. The likelihoods sum up to 1 if the background is included. By default, however, if the likelihood of belonging to one class is larger than 50% then this pixel is said to belong to the specific class class. Three different thresholds were considered (i.e 50%, 25%, and 12.5%). The threshold selection was based on testing on the training data and no adjustments were made based on the test data.

#### Selection of the n-largest object

The most frequently used post processing was selection of the n largest object(s). This was used for applications where we from anatomical a priori information only expect one or two contiguous objects. For instance we expect to find at most two eyes and thus use this setting to remove small outlier objects. Object connectivity was computed using a standard 26-connectivity.

#### Touch other objects

The generic post processing allows to require that objects touch each other to be included. The setting of this parameter was decided from anatomy, the trachea is the only one of the classes that is required to touch another object (parenchyma).

### Number of training data required

In order to test the limits of how few subjects are needed to achieve good results, multiple models were trained for the skeletal structure application. Using training set sizes of 3, 5, 10, 20, and 40 subjects, five different models were created and tested on the same ground truth data set (n=13). A comparison of patient-level performance between the networks were performed and specific cases were also evaluated to determine the effect of being included in the training data. This was achieved by comparing the n=3 model and the n=40 model on a on a per-patient basis using Jaccard scores. Expanding on this topic, it was examined for which cases the Jaccard score was improved the most, hypothesising that an increase in Jaccard score after an increase in training data size depend on initial score.

To explore the generalizability of the model, a scan of a patient’s knee was included in the test set whereas knees were on purpose excluded from the training data set. Thus, the model had to perform inference without prior exposure to this type of anatomy, which is known as ’zero-shot’ inference.

### Model evaluation and statistical analysis

#### Volume-based analysis

Dice and Jaccard scores were calculated according to Eqs. 1 and 2.

1 $$\begin{aligned} \text {Dice Score} = \frac{2TP}{2TP + FP + FN} \end{aligned}$$

2 $$\begin{aligned} \text {Jaccard Score, IoU} = \frac{TP}{TP + FP + FN} \end{aligned}$$

where $TP$ refers true positive and $FN$ to false negative. In the case of segmentation we define a true positive value to be a correctly labeled voxel.

#### Surface-based analysis

In the surface-based analysis, the exterior surfaces of both the ground truth and network-segmented models were determined. For every point on the ground truth surface, the distance to the nearest point on the network-segmented surface was computed utilizing triangulation. Subsequently, the evaluation was conducted on the 95th percentile of both signed and absolute surface distances. The sign of the distances was derived from the normal vector of the ground truth surface. A value of, for instance, 1 mm should be interpreted as “for 95 percent of surface, the spatial deviation between the ground truth segmentation and the automatic process does not exceed 1 mm”.

#### Failure mode analysis

In order to investigate potential causes of poor segmentation we looked at data sets with the lowest Dice score for each of the six tasks. 

## Results

**Fig. 8:**
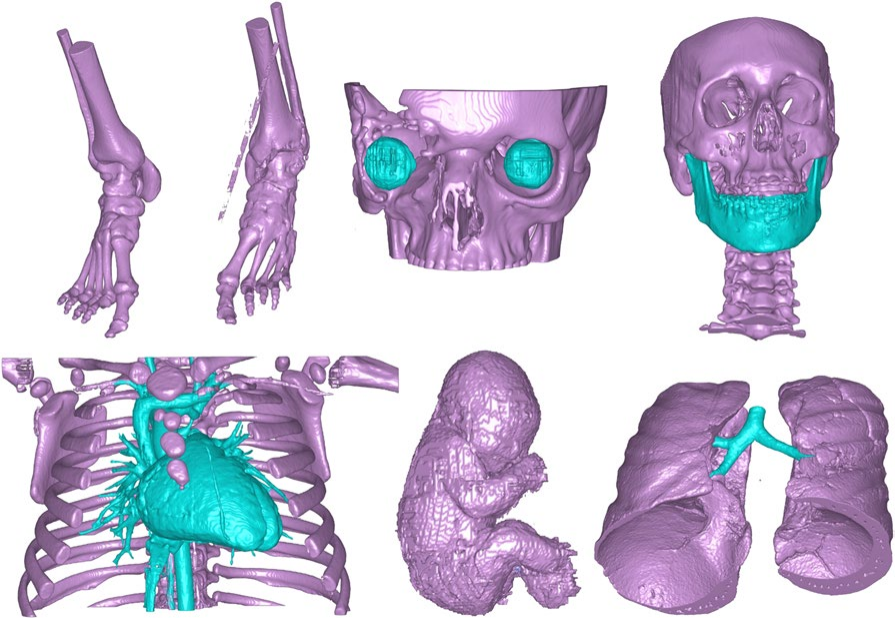
Visualization of median performing segmentations

**Fig. 9:**
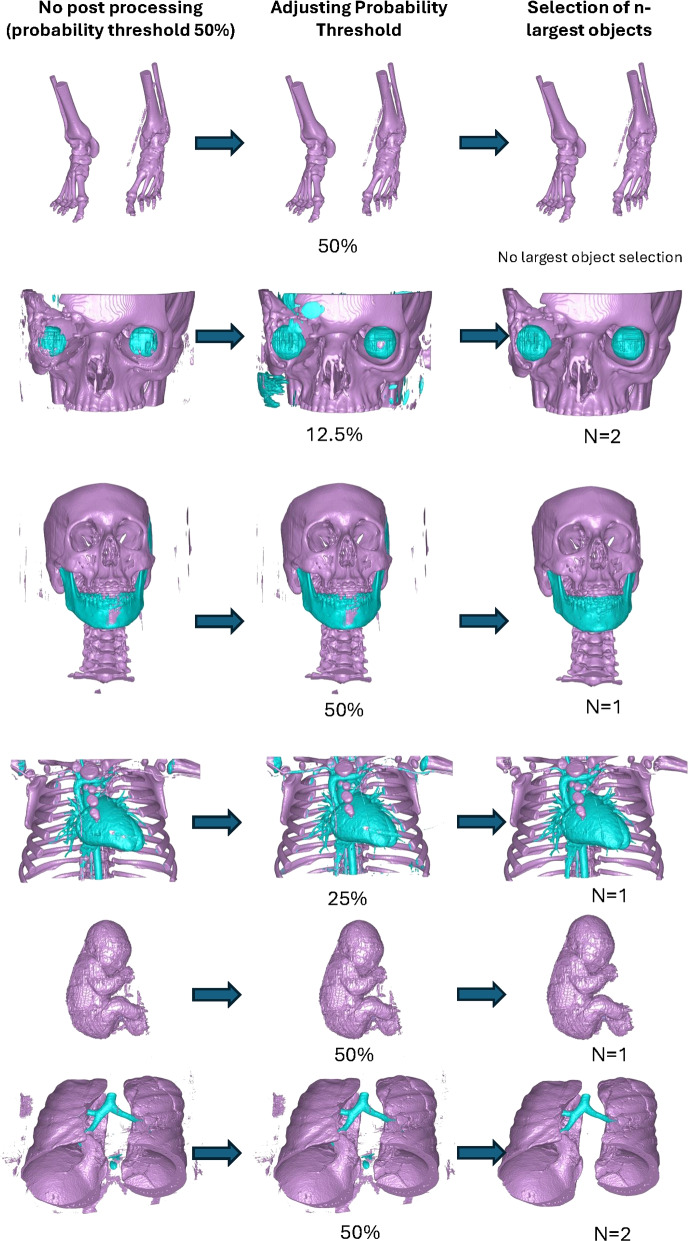
Steps of the post processing procedure

**Fig. 10:**
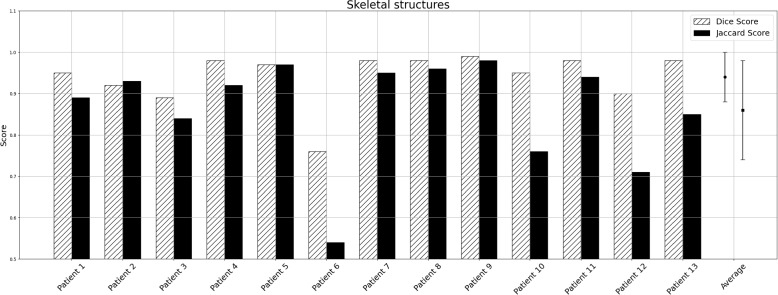
Per-patient performance for skeletal structure segmentation. Striped bars show Dice score and solid bars show Jaccard score

**Fig. 11:**
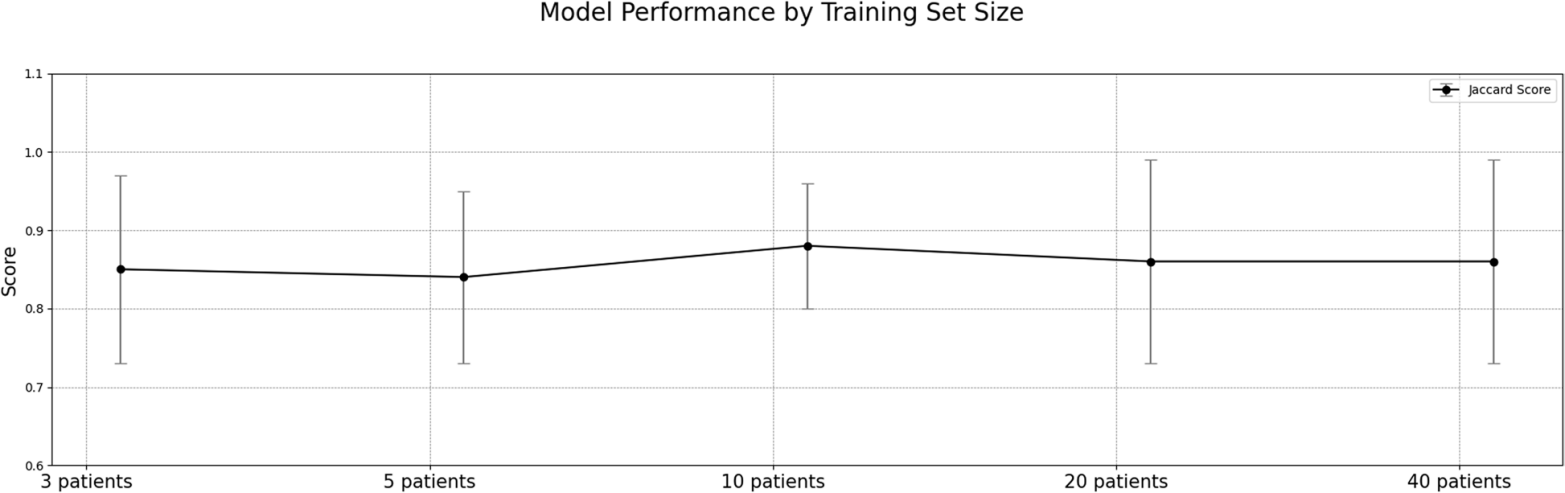
Performance versus number of cases used for training

**Fig. 12:**
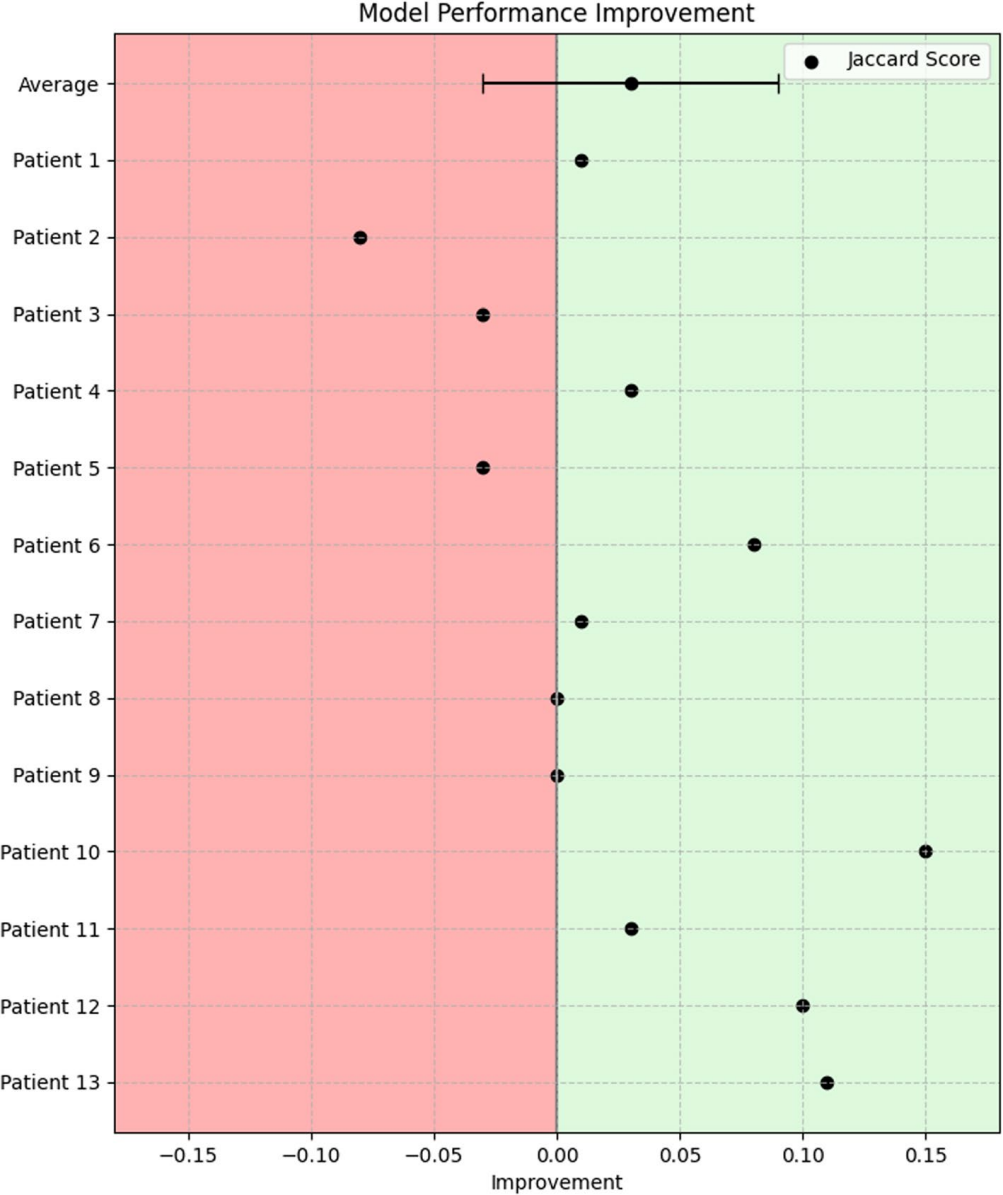
Difference between model trained with 40 patients, "n=40 model", versus model trained using 3 patients, "n=3 model". Positive values show higher performance when trained with more training data

**Fig. 13:**
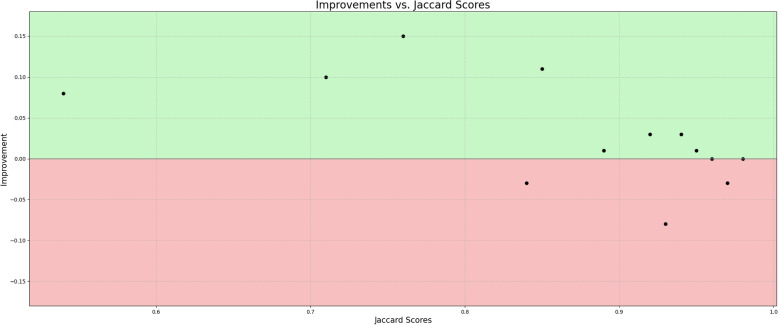
Improvements vs. Jaccard score. X-axis show Jaccord score for "n=40 model" and the y-axis the difference between "n=40 model" versus the "n=3 model". Positive values is indicative of higher performance when trained with more training data

**Fig. 14:**
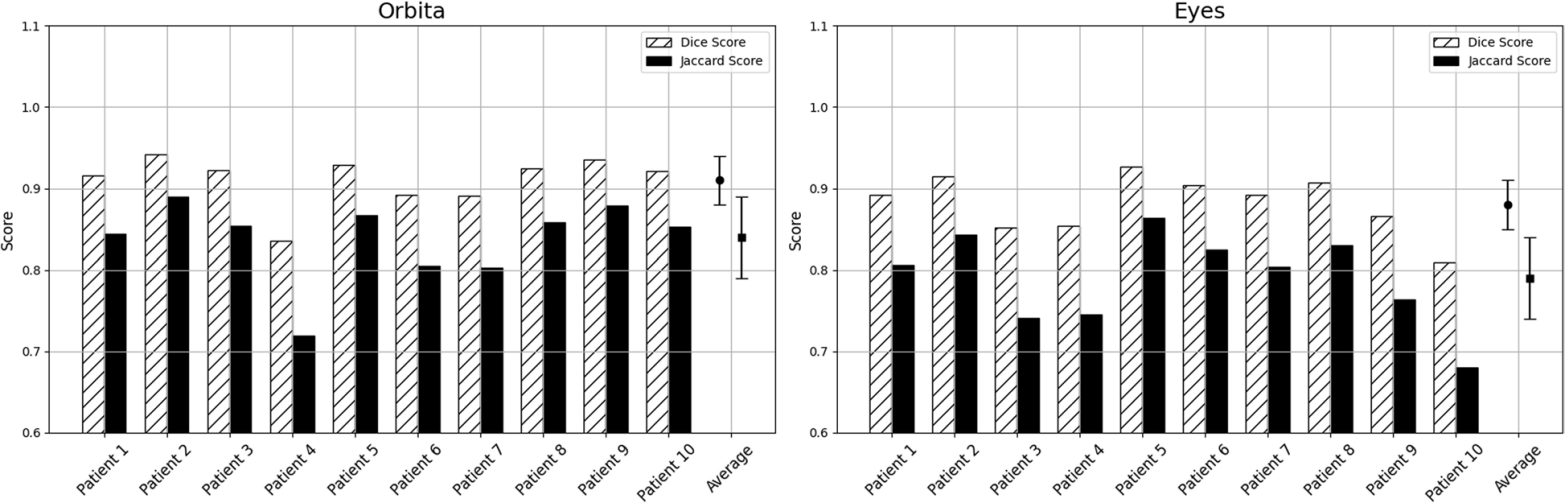
Per-patient performance on orbital segmentation. Left panel shows results for the class *orbita,* and right panel shows the results for the class *eyes.* Note that low scores are not consistent between patients, such as for patient #3 there is a low score for the class *orbita,* whereas for patient #6 there is a low score for the class *eyes*

**Fig. 15:**
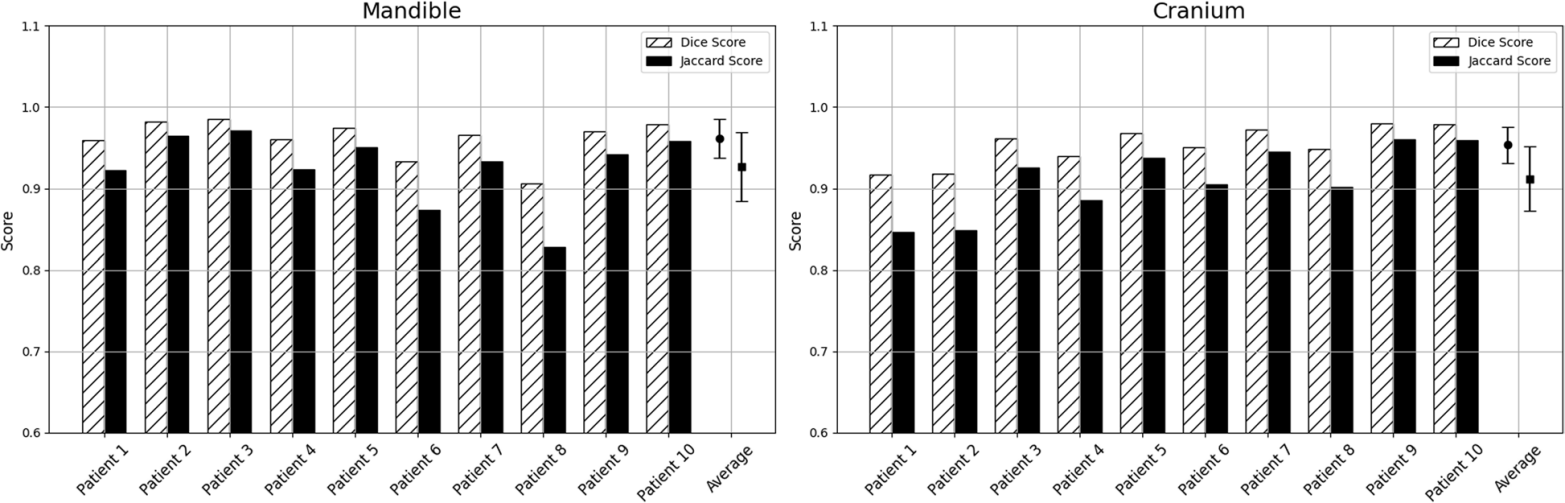
Per-patient performance on mandibulo-cranial structure segmentation. Left panel shows the result from the class *mandible*, right panel shows the result from the class *cranium*

**Fig. 16:**
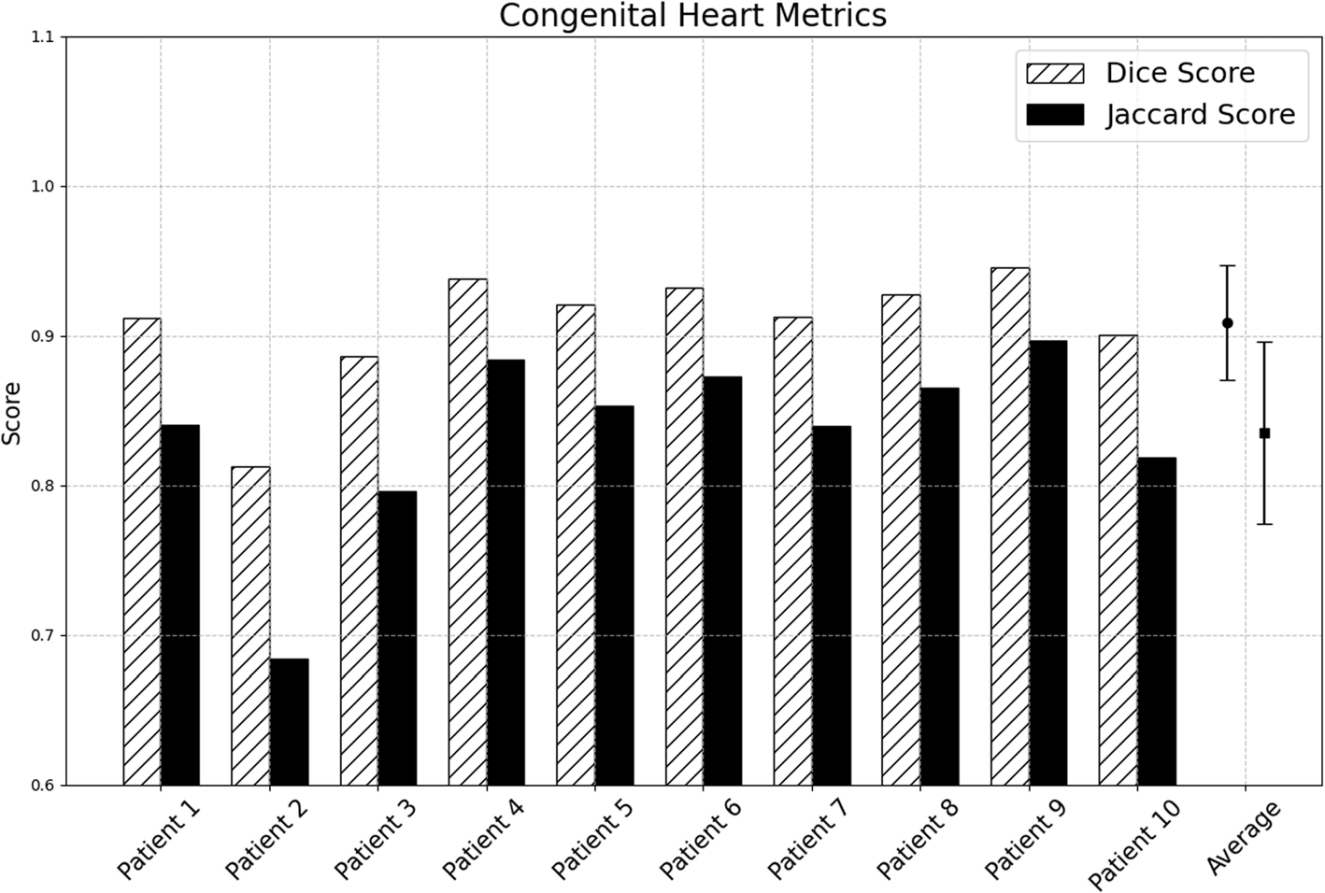
Per-patient performance for congenital heart defects. Striped bars show Dice score and solid bars show Jaccard score

**Fig. 17:**
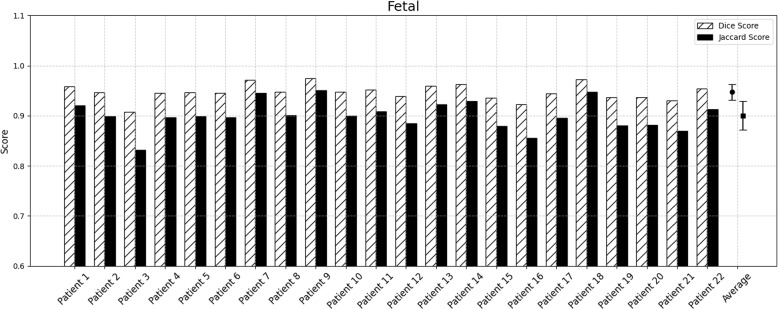
﻿Per-patient performance for fetal anatomy. Striped bars show Dice score and solid bars show Jaccard score

**Fig. 18:**
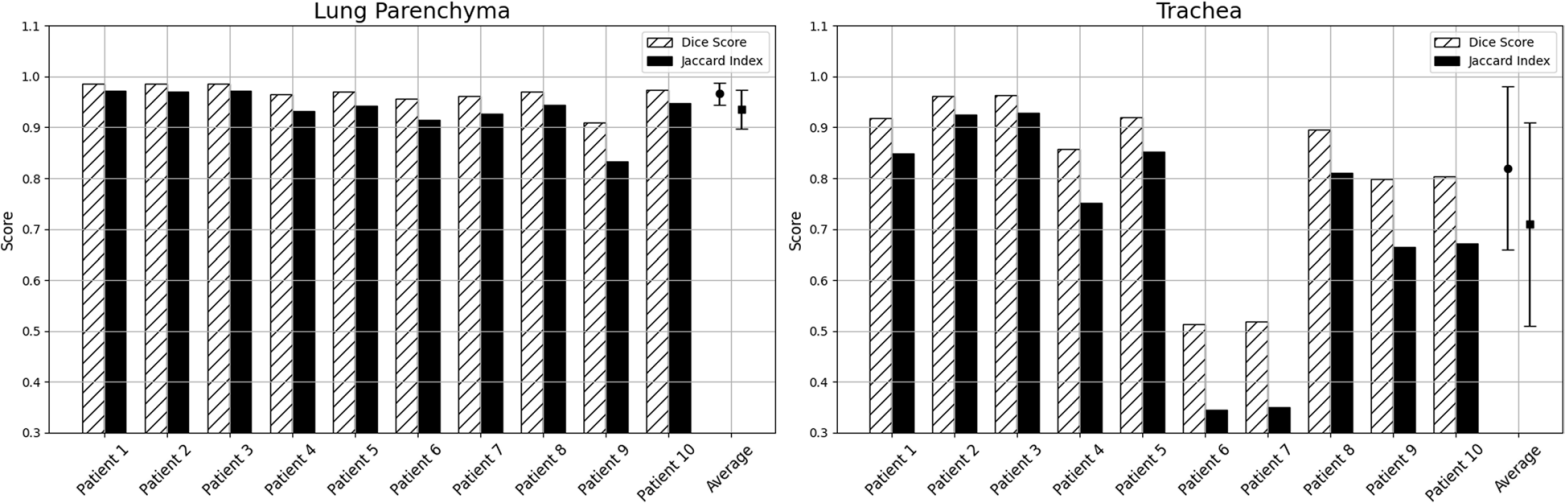
﻿Per-patient performance for pulmonary-tracheal structures. Striped bars show Dice score and solid bars show Jaccard score

**Fig. 19:**
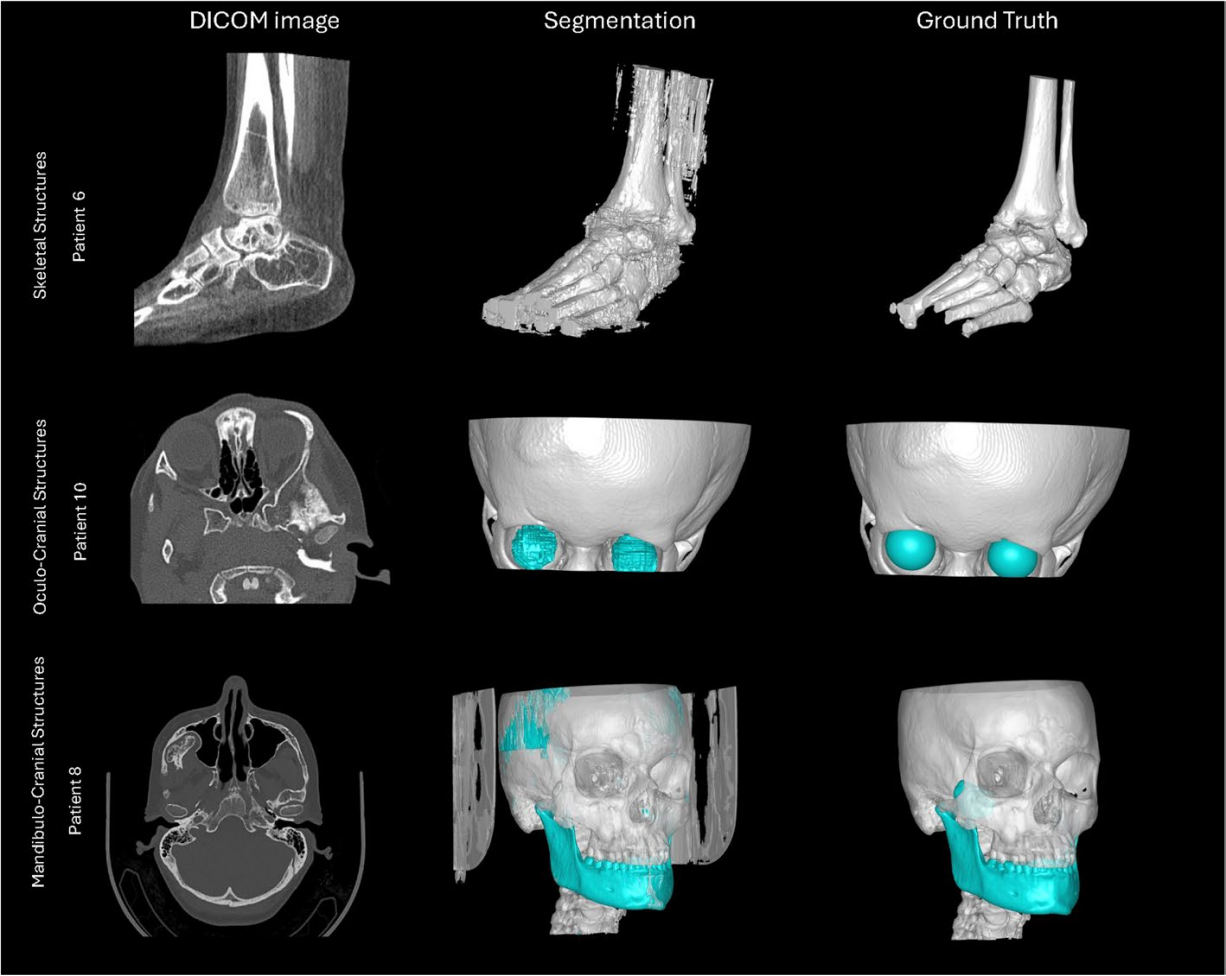
﻿Lowest scoring segmentations in the categories Skeletal Structures, Oculo-Cranial Structures and Mandibulo-Cranial Structures. Left column shows a representative DICOM slice, middle column shows automated segmentation results including post-processing, and the right column shows the manual segmentation

**Fig. 20:**
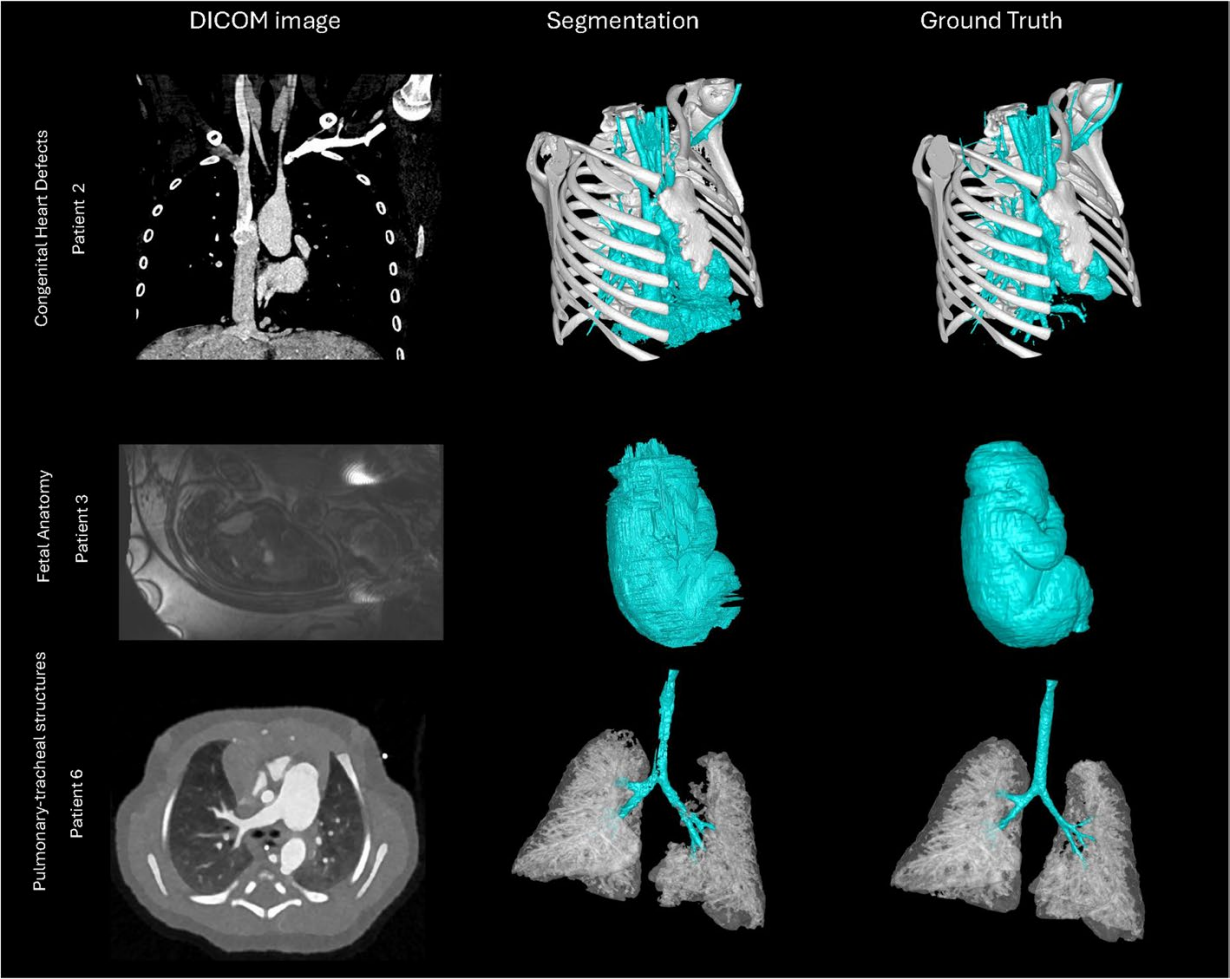
Lowest scoring segmentations in the categories Congenital Heart Defects, Fetal Anatomy and Pulmonary-tracheal Structures. Left column shows a representative DICOM slice, middle column shows automated segmentation results including post-processing, and the right column shows the manual segmentation﻿

Visualization of median performing segmentations is shown in Fig. 8. Steps of the post processing procedure is shown in Fig. 9. Results of network inference on test data for the different clinical use cases can be seen in Table 5 and in in Figs. 10, 11, 12, 13, 14, 15, 16, 17 and 18. On average, the networks segmented a 256x256x256 pixel volume in 10.10 s on a gaming laptop (Alienware, NVIDIA Ti 3080 GPU). For the skeletal segmentation task, five separate models were trained on different sizes of training set. Results from these tests are shown in Table 6 and in Fig. 11. Figure 12 shows the calculated difference of the model trained on three patients versus the model trained on forty patients, of which the smaller training set was part of the larger. Which patients in the test data that benefited the most by expanding the training set was analysed by evaluation of improvement in Jaccard score, shown in Fig. 13. Furthermore, a zero shot analysis was performed on a specific patient from this orthopedic use case, namely patient 10 (see Fig. 10). This zero shot analysis yielded Dice score of 0.95 and Jaccard score of 0.91. Figures 19 and 20 illustrates the resulting segmentations of the lowest scoring segmentations.

**Table 5:** Evaluation metrics

Structure	Dice Score $\varvec{\pm }$ SD	Jaccard Score $\varvec{\pm }$ SD	95%-tile dist. (mm) $\varvec{\pm }$ SD	Median dist. (mm) $\varvec{\pm }$ SD
Skeletal Structures	0.94 ± 0.06	0.89 ± 0.10	0.75 ± 0.76	0.24 ± 0.23
Oculo-Cranial Structures				
Orbita	0.91 ± 0.03	0.84 ± 0.05	1.06 ± 0.82	0.30 ± 0.16
Eyes	0.88 ± 0.03	0.79 ± 0.05	2.08 ± 0.67	0.65 ± 0.21
Mandibulo-Cranial Structures				
Mandible	0.96 ± 0.02	0.93 ± 0.04	0.69 ± 0.76	0.13 ± 0.04
Cranium	0.95 ± 0.02	0.91 ± 0.04	0.56 ± 0.22	0.12 ± 0.06
Congenital Heart Defects	0.91 ± 0.04	0.84 ± 0.06	11.07 ± 8.16	0.17 ± 0.08
Fetal Anatomy	0.95 ± 0.02	0.90 ± 0.03	4.65 ± 1.70	1.17 ± 0.34
Pulmonary-tracheal structures				
Parenchyma	0.97 ± 0.02	0.94 ± 0.04	1.00 ± 1.09	0.19 ± 0.08
Trachea	0.82 ± 0.16	0.71 ± 0.20	3.71 ± 5.42	0.31 ± 0.22

**Table 6:** Evaluation metrics of skeletal structure application trained with different training data set sizes

Number of Patients	Dice score $\varvec{\pm }$ SD	Jaccard score $\varvec{\pm }$ SD	95-%tile dist. (mm) $\varvec{\pm }$ SD	Med. dist. (mm) $\varvec{\pm }$ SD
3	0.92 ± 0.08	0.86 ± 0.12	0.78 ± 0.78	0.27 ± 0.24
5	0.92 ± 0.07	0.85 ± 0.11	0.98 ± 1.26	0.26 ± 0.22
10	0.94 ± 0.05	0.89 ± 0.08	0.73 ± 0.76	0.25 ± 0.22
20	0.93 ± 0.09	0.87 ± 0.13	0.79 ± 0.91	0.23 ± 0.23
40	0.94 ± 0.06	0.89 ± 0.10	0.75 ± 0.76	0.24 ± 0.23

## Discussion

The proposed framework demonstrates high medical image segmentation performance across various tasks despite being trained on a relatively small number of subjects. Evaluation metrics in Table 5 show the performance of models trained on different clinical use cases. Even though the number of subjects used for training was merely in the tens, the models achieved an average Dice score of 0.92 (SD = ±0.06) and a Jaccard score of 0.86 (SD = ±0.08) over all cases.

It is likely that with different architectures and tuning of hyper-parameters a slightly increased result could be achieved. However, a slight improvement in performance may be of little clinical relevance. What is of clinical relevance though, is that the accuracy is high enough to translate to significant time savings. This also puts an emphasis in the interaction with an automated segmentation algorithm and efficient tools for manual corrections. Furthermore, it is important that the number required subjects are low so that software developers with reasonable effort can add new models. Specifically, in field of clinical 3D printing there is a long and growing list of applications which each ideally should have their own specific segmentation algorithm to save time and work in the clinical routine.

### Interpretation of segmentation metrics

There are numerous challenges in interpreting segmentation metrics, as demonstrated in the following consensus paper on the subject [15]. In the present study we chose to include Dice score, Jaccard score and surface distance measurements, given their widespread usage in existing literature. However, it is important to highlight that in a clinical setting, the emphasis should lie on the application at hand, such as swiftly generating high-quality anatomical models for 3D printing or virtual planning of a surgical procedure rather than achieving a specific segmentation performance score. Segmentation errors, such as incomplete bone filling or incorrect inclusion of left/right markers in the segmentation, may lead to notable penalties. Such inconsistencies may typically be easily fixed with minor manual editing.

The distance measurements presented in Table 5 relates to the distance difference between the surface of the automated segmentation and ground truth segmentation. All models achieved low average median distance meaning the automated segmentation coincides well with the manual segmentation. The 95th percentile distance is indicative of the model’s potential limitations, with the understanding that 95% of the data points fall below this measured distance, highlighting the upper boundary of error in the model’s predictions. This measure may be problematic in the case where, for instance, a detail such as a smaller vessel may be missed in the automatic segmentation, whereas it is present in the ground truth. This is the case in the congenital heart defect segmentation, where the segmentation often miss small vessels in the lungs that does not have any clinical significance.

### Interpretation of results

In relation to this study, we therefore urge that the Dice and Jaccard scores be interpreted cautiously. The metrics, while widely used, can be influenced by factors such as cropping and post-processing, both of which we employed in this study See Fig. 9. Although these measures offer a standardized evaluation method, our primary goal was not to achieve high segmentation scores per se. Rather, we aimed to demonstrate that even with limited training data, it is possible to achieve clinically useful segmentation results in a 3D printing context. Therefore, while Dice and Jaccard scores are reported for comparability, our focus remains on practical segmentation performance for 3D printing applications, rather than on optimizing these metrics alone. Furthermore, our results merely reflects a single training and it recently has been shown that repeated training with the exact same data can generate statistically different models [16].

Moreover, segmentation performance of bones and soft tissue organs such as the pancreas or liver should not be directly compared as they present different levels of segmentation difficulty. Most of the examples used in this study comprise structures in IV-contrast settings, however, also included in the study were non-bone, non-contrast enhanced structures - specifically eye-class in the Oculo-Cranial Structures category and fetuses in the Fetal anatomy category. These categories are more complex structures, albeit with greater boundary contrast than soft-tissue organs as they are enclosed within cavities that provide clear anatomical boundaries.

The structures used in this study suggest that the networks learn a combination of intensity, local structure, and texture features to classify voxels into appropriate structures. This is evident, for example, in the congenital heart disease segmentation task, where the network successfully distinguishes bones from contrast-enhanced vessels, even though these structures overlap completely in intensity. Such separation would only be possible through learning a combination of intensity and local texture. Another example is the network’s ability to segment and fill bone structures, which is not possible using only intensity thresholding.

Importantly, we do not expect the networks in our setup to learn specific anatomical relationships, as these are likely lost in the augmentation process. While not learning anatomy could be seen as a drawback, in a 3D printing context, it may actually be an advantage since the applications often involve grossly abnormal anatomy. For instance, our test data include cases of severe scoliosis and complex congenital heart defects, such as heterotaxy syndrome.

### Generalizability

A model with excellent generalizability gives quantitatively the same performance regardless of which case it is used on. We can see that the generalizability of the models produced by the framework is good as the results are consistently good for all subjects in the test cases. For instance the model achieved good scores in segmentation of a knee even though the model had not been trained on any knee. This indicates that rather than learning anatomy, the models have learned how to locally classify tissue types based on texture and pixel intensities. This an important trait that allows the framework produce models that are able to handle a large variability in anatomy such a broken orbital floor or vessels in the case of congenital heart defects. Although the knee case was used to highlight this fact, testing the model trained on three subjects (’n=3 model’) is essentially a zero-shot analysis. The model was trained using data that included only arms, pelvis, and feet, along with parts of the tibia and fibula, and was presented with anatomies it has not encountered before.

Although the generalizability of the framework is good, it is not perfect. In order to understand limitations, generalizability and potential failure modes, we investigated the patients where we got the lowest Dice score for each application (Figs. 19 and 20).

- In the orthopedic test data with the lowest Dice score we can see that there is a somewhat challenging image quality in combination with poor bone quality that likely explains the poor result. For the orbital test data with the lowest Dice score we can see that it is challenging to see the eyes, in combination that the volume is cropped and do not fully cover the left eye.
- In the mandible test data with the lowest Dice score we can see that for parts of the skull have been incorrectly classified as mandible. We can also see that the part of a skull support is segmented as skull. In addition, when reviewing the image closely, the right condyle contains a bony-mass that is not segmented.
- In the cardiac test data with the lowest Dice score the liver is unusually contrast filled and incorrectly included in the automatic segmentation. There are also some smaller vessels that are missing.
- In the fetal dataset with the lowest Dice score the image quality is difficult and specifically there are significant motion artifacts in the head region.
- In the lung case with the lowest Dice score the HU units are outside normal range as the images is from a new born baby only hours old. In addition, there is a contrast filled catheter in the esophagus that may confuse the network.

In summary, these segmentations were harder to perform due to one or more out of the following possible reasons:

- low image quality or imaging artifacts,
- image properties are different to the training data such as image resolution, image intensity, image contrast,
- the input volume is significantly outside of the domain of the training data such as new anatomy, significant pathology that alters the image, or contrast agent differences.

One of the general approaches to tackle limited generalizability in deep learning is to add more training data. Here, the results from the skeletal structure use case, where 5 networks were trained with different amount of training data, provide some insight. Interestingly, the network trained with only 3 (“n=3 model”) subjects achieved a good score not far off the results from the network trained with 40 subjects (“n=40 model”). The results is shown in Table 6 and Fig. 11. The scores were relatively constant with only 2 percent difference in average dice score between “n=3 model” and “n=40 model”. The models are compared on a per-patient basis in Fig. 12.

The “n=40 model” performed better on patients #6, 10, 12 and 13. These patients were the patients where the initial model struggled to segment as shown in Fig. 10. This points to the fact that the increase of performance is most prominent on the cases that are the hardest to segment to begin with. This is further illustrated in Fig. 13 where on the x-axis the Jaccard score for the “n=40 model” and on the y-axis is the increase in performance between “n=40 model” and “n=3 model”. The “n=3 model” slightly outperformed the “n=40 model” for three patients in particular patient #2 (Fig. 12). This highlights a trade-off between generalizability and performance and suggests that adding data to the training set may not consistently enhance performance across all test objects in the test set.

The implication is that if a user wants to segment challenging cases, an expansion of the training set with challenging cases could be beneficial. However, if the user wishes to segment easier cases, not much improvement could be expected from expanding the data set. Adding too many simple cases might in fact reduce the performance in challenging cases. What specific characteristics constitute easy and challenging cases are typically unknown and likely problem specific.

From the users point of view, in a clinical setting it might be better to have multiple algorithms for specific use cases, such as one algorithm trained with contrast on board and one without contrast on board for the same anatomical region rather than having one sub-optimal algorithm. Furthermore, the models can improve as new data is made available. In extension this also relates to the requirements set by the intended end use case, such as surgical guides for orthopedics or preoperative planning for congenital heart defect surgeries. Future investigations may include determining what training data should be included to achieve higher performance at particular segmentation tasks.

## Limitations

The fixed hyperparameters might not be optimally suited for every specific case, potentially leading to sub-optimal results in some scenarios. Further research could explore balancing adjustable hyper-parameters as well as including the parameters in the training.

Except for the fetal anatomy network where 5-fold cross-validation was used, the models were trained only once. It is known that the randomness involved in the training process can give improved results just by retraining the model.

Further investigations should include cases such as soft tissue organs (pancreas, liver, kidneys etc.) where the delineation of target organ and surrounding tissue may be not as clear as the structures used in this paper.

As measuring Dice score, Jaccard score and surface distances are the most commonly used evaluation metrics, the same methodology was used here. Further work could include quantitative and qualitative user metrics for individual use cases. Some studies validate model performance by comparing timings of manual versus automatic segmentation [16].

### Implications

The presented 3D deep learning segmentation framework alleviates many of the challenges in applied medical image segmentation. Especially the ability to achieve high accuracy using limited number of training subjects addresses a significant bottleneck in providing clinically relevant segmentation algorithms. As the high quality results was archived across diverse organs and tissues with little to no tuning of hyper parameters we eliminate the need for developing specialized training pipelines for each application.

The key implication of the framework is its potential to accelerate and streamline clinical workflows. By reducing the data and computational requirements, the approach significantly lowers the entry barriers for adoption AI-driven segmentation techniques. Adding a new application essentially becomes a plug-and-play process where all that is needed is a small set of training data that is in the order of tens of subjects. Subsequently, it becomes possible to develop and train dedicated segmentation algorithms for niched clinical applications such as 3D printing.

## Conclusion

In this study, we introduced a deep learning framework for general 3D medical segmentation. The framework demonstrated high performance across a spectrum of clinical use cases, showing promise in automating medical image segmentation tasks. By circumventing the need for large training datasets and powerful computational resources, our approach offers an avenue for easier adoption within healthcare systems.

## Supplementary Information

Supplementary Material 1.

## Data Availability

Data cannot be made freely available to the public, due to privacy regulations and informed consent.

## Abbreviations

2D: 2 dimensional
3D: 3 dimensional
CNN: Convolutional neural network
CT: Computed tomography
GPU: Graphical processing unit (graphics card)
MR: Magnetic resonance
ML: Machine learning

## Footnotes

1 The fetal application used slightly different hyper-parameters as this was the original application and we chose not to change the parameters based on our original publication.
