# Supplementary material for: Generalizable deep learning framework for 3D medical image segmentation using limited training data
Source: 3D Print Med. 2025 Mar 6;11:9. doi: 10.1186/s41205-025-00254-1 (PMC11884210; doi:10.1186/s41205-025-00254-1)
Supplement: Supplementary file 1 — Supplementary Material 1. [file 41205_2025_254_MOESM1_ESM.zip › Supplemental material - Test data.pdf]

Skeletal structures

Image	Usage	Description	AcquisitionDat	Age (year)	Sex	Scanner	Resolution (mm)	SizeX	SizeY	SizeZ
Image 1	TEST	Spine	Unknown	1	F	Philips	0,30	307	307	723
Image 2	TEST	Hand positionned over the chest. Sever joint disorder	2021	61	F	Siemens	0,52	511	507	298
Image 3	TEST	Hand data set with ulna and radius	2020	15	F	GE	0,81	278	512	426
Image 4	TEST	Skull with defect	2018	74	M	Philips	0,43	512	512	399
Image 5	TEST	Skull with defect	2019	32	F	Siemens	0,43	512	512	370
Image 6	TEST	Feet	2020	34	F	Siemens	0,48	314	247	368
Image 7	TEST	Pediatric tumor with contrast on board left	2020	0,4	F	Siemens	0,37	349	369	444
Image 8	TEST	Feet	2018	22	F	Siemens	0,61	512	512	355
Image 9	TEST	Semi-thorax (neonate)	2021	0,06	M	Siemens	0,27	399	185	346
Image 10	TEST	Young pelvis female	2020	2	F	Philips	0,31	339	651	474
Image 11	TEST	Young pelvis male	2019	1	M	Siemens	0,34	512	512	324
Image 12	TEST	Knee with contrast on board left	2017	61	M	Siemens	0,79	254	200	310
Image 13	TEST	Severe scoliosis lower part	2019	11	F	Philips	0,49	512	512	344
Image 14	TEST	Pretty severe scoliosis	2019	14	F	Toshiba	0,50	512	512	919
Image 15	TEST	Knees	2022	38	F	Toshiba	0,73	512	512	384

Oculo-cranial structures

Image	Usage	Description	AcquisitionDat	Age (year)	Sex	Scanner	Resolution (mm)	SizeX	SizeY	SizeZ
Image 1	TEST	Candidaet for maxilla reposition	Unknown	35	F	GE	0,31	196	368	235
Image 2	TEST	Cranioplasty	Unknown	38	M	Philips	0,43	441	369	238
Image 3	TEST	Orbita floor fracture	2021	82	F	Siemens	0,54	250	288	154
Image 4	TEST	Orbita floor fracture	2021	51	F	Siemens	0,56	256	268	179
Image 5	TEST	Cranioplasty, including hemi orbita fracture	2019	80	F	Philips	0,43	263	344	224
Image 6	TEST	Cranioplasty	2018	94	F	Philips	0,43	281	453	211
Image 7	TEST	Major skull trauma part of orbita missing	2023	14	M	Philips	0,47	224	290	191
Image 8	TEST	Major skull trauma part of orbita missing, multiple metal plates	2023	29	M	Philips	0,43	154	336	241
Image 9	TEST	Major skull trauma part of orbita missing	2023	23	M	GE	0,49	212	268	226
Image 10	TEST	Fibrotic dysplacia	2019	4	M	Philips	0,43	306	329	172

Mandibulo-cranial structures

Image	Usage	Description	AcquisitionDat	Age (year)	Sex	Scanner	Resolution (mm)	SizeX	SizeY	SizeZ
Image 1	TEST	Orbita floor fracture	2021	82	F	Siemens	0,54	279	349	250
Image 2	TEST	Orbita floor fracture	2021	22	M	Siemens	0,39	372	389	373
Image 3	TEST	Candidate for ortognatic surgery, metal braces	Unknown	22	F	Philips	0,43	370	317	273
Image 4	TEST	Normal anatomy, skull defect from anatomy outside region	2023	35	F	GE	0,31	512	512	516
Image 5	TEST	Candidate for ortognatic surgery	2021	37	M	Siemens	0,37	512	512	439
Image 6	TEST		2023	46	F	Siemens	0,37	512	512	425
Image 7	TEST		Unknown	35	F	Siemens	0,43	512	512	453
Image 8	TEST		2023	54	F	Philips	0,43	512	512	452
Image 9	TEST	Candidate for cranioplasty	2024	36	M	GE	0,50	489	369	444
Image 10	TEST		2023	22	M	Philips	0,43	512	512	469

efects

Image	Usage	Description	AcquisitionDat	Age (year)	Sex	Scanner	Resolution (mm)	SizeX	SizeY	SizeZ
Image 1	TEST		Unknown	23	M	Siemens	0,35	512	512	340
Image 2	TEST		2023	15	F	Siemens	0,50	432	565	528

Congenital heart de											
	Image 3	TEST		2023	Unknown	M	Siemens	0,20	512	512	328
	Image 4	TEST		2023	0,083	M	Siemens	0,34	360	512	363
	Image 5	TEST		2022	0,42	F	Siemens	0,30	508	512	328
	Image 6	TEST		2023	0,67	M	Siemens	0,34	452	512	337
	Image 7	TEST		2023	0,25	M	Siemens	0,33	314	501	345
	Image 8	TEST		2023	0,0027	M	Siemens	0,25	512	512	398
	Image 9	TEST		2023	12	F	Siemens	0,46	512	512	281
	Image 10	TEST		2023	0,67	M	Siemens	0,32	512	512	353

Fetal anatomy	Image	Usage	Description	AcquisitionDat	Age (year)	Gestational ag	Sex	Scanner	Resolutior	SizeX	SizeY	SizeZ
	Image 1	TEST		2021	36	39,00	F	Siemens	1,56	256	256	184
	Image 2	TEST		2021	34	34,57	F	Siemens	1,56	256	256	184
	Image 3	TEST		2017	Unknown	37,57	F	Siemens	0,78	512	416	308
	Image 4	TEST		2021	36	38,57	F	Siemens	1,56	256	256	184
	Image 5	TEST		2021	30	36,57	F	Siemens	1,56	256	256	184
	Image 6	TEST		2021	33	37,00	F	Siemens	1,56	256	256	184
	Image 7	TEST		2016	Unknown	37,00	F	Siemens	0,78	512	336	307
	Image 8	TEST		2021	25	35,14	F	Siemens	1,56	256	256	184
	Image 9	TEST		2020	Unknown	36,86	F	Siemens	0,96	380	416	266
	Image 10	TEST		2021	32	36,00	F	Siemens	1,56	256	256	184
	Image 11	TEST		2021	35	33,29	F	Siemens	1,56	256	256	184
	Image 12	TEST		2021	30	36,86	F	Siemens	1,56	256	256	184
	Image 13	TEST		2021	26	34,86	F	Siemens	1,56	256	256	184
	Image 14	TEST		2020	Unknown	37,00	F	Siemens	0,78	512	336	410
	Image 15	TEST		2021	30	35,29	F	Siemens	1,56	256	256	184
	Image 16	TEST		2021	30	Unknown	F	Siemens	1,64	256	256	176
	Image 17	TEST		2021	31	36,29	F	Siemens	1,95	256	256	164
	Image 18	TEST		2016	Unknown	37,14	F	Siemens	0,78	512	336	307
	Image 19	TEST		2021	41	38,71	F	Siemens	1,56	256	256	184
	Image 20	TEST		2021	39	38,71	F	Siemens	1,68	256	256	171
	Image 21	TEST		2021	28	37,57	F	Siemens	1,56	256	256	184
	Image 22	TEST		2021	42	32,29	F	Siemens	1,56	256	256	184

Pulmonary-tracheal structures	Image	Usage	Description	AcquisitionDat	Age (year)	Sex	Scanner	Resolution (mm)	SizeX	SizeY	SizeZ
	Image 1	TEST	Tumor on trachea (no contrast)	2022	53	F	GE	0,86	512	512	538
	Image 2	TEST	Complete spine, lung cancer, no contrast on board	1993	76	F	Siemens	0,53	512	512	533
	Image 3	TEST	Complete spine, lung cancer, no contrast on board	1992	60	F	Siemens	0,56	512	512	521
	Image 4	TEST	Pediatric aortic coarctation (minimum contrast)	2018	8	M	Unknown	0,68	311	406	351
	Image 5	TEST	Complex congenital heart disease, large and dilated heart (contrast)	2023	16	M	Siemens	0,45	539	725	730
	Image 6	TEST	Congenital heart disease	2021	2	F	Siemens	0,42	512	510	378
	Image 7	TEST	Aortic arch ring (contrast)	2023	0,003	F	Siemens	0,22	480	512	400
	Image 8	TEST	Atrial septal defect and pacemaker (contrast)	Unknown	14	F	Siemens	0,57	420	512	268
	Image 9	TEST	Aortic coarctation and abnomal carotids (contrast)	2023	0,4	F	Siemens	0,33	512	512	415
	Image 10	TEST	Slow growing endocrine cardiac tumor (contrast)	2019	56	F	Siemens	0,60	512	512	463
