# Supplementary material for: Generalizable deep learning framework for 3D medical image segmentation using limited training data
Source: 3D Print Med. 2025 Mar 6;11:9. doi: 10.1186/s41205-025-00254-1 (PMC11884210; doi:10.1186/s41205-025-00254-1)
Supplement: Supplementary file 1 — Supplementary Material 1. [file 41205_2025_254_MOESM1_ESM.zip › Supplemental material - Train data.pdf]

Skeletal structures

Image	Usage	Description	AcquisitionDate	Age (year)	Sex	Scanner	Resolution (mm)	SizeX	SizeY	SizeZ
Image 1	TRAIN	Both arms (elbow to metacarpals)	Unknown	14	F	Toshiba	0,54	512	512	541
Image 2	TRAIN	Part of hands, ulna and radius	Unknown	46	F	Toshiba	0,45	512	512	267
Image 3	TRAIN	Spine, heavy lung tumour	1992	34	F	Siemens	0,55	512	512	494
Image 4	TRAIN	Lumbar pelvis, Massive calcification aorta	Unknown	80	F	Siemens	0,69	512	512	563
Image 5	TRAIN	Spine, small amount of contrast agent	Unknown	42	F	Siemens	0,65	512	512	270
Image 6	TRAIN	Spine, contrast agent in blood	1993	76	F	GE	0,53	512	512	533
Image 7	TRAIN	Spine	Unknown	70	M	Siemens	0,72	512	512	190
Image 8	TRAIN	Spine	Unknown	60	F	Siemens	0,56	512	512	521
Image 9	TRAIN	Skull	Unknown	21	M	Philips	0,43	512	512	405
Image 10	TRAIN	Spine	Unknown	52	M	Siemens	0,98	347	289	190
Image 11	TRAIN	Left hand	2022	62	F	Siemens	0,29	512	512	369
Image 12	TRAIN	Right hand	2022	62	F	Siemens	0,29	512	512	378
Image 13	TRAIN	Arms	2022	53	F	Siemens	0,74	512	512	425
Image 14	TRAIN	Foot	Unknown	61	M	Siemens	0,78	313	221	261
Image 15	TRAIN	Foot	Unknown	34	F	Siemens	0,53	412	295	317
Image 16	TRAIN	Right hand	2021	56	Unknown	Toshiba	0,63	213	243	644
Image 17	TRAIN	Part of right hand, ulna & radius	2018	27	Unknown	Philips	0,45	286	350	648
Image 18	TRAIN	Left Hand	2019	19	Unknown	Philips	0,40	304	301	896
Image 19	TRAIN	Part of left hand, ulna & radius	Unknown	27	Unknown	Philips	0,45	288	349	648
Image 20	TRAIN	Part of right hand, ulna & radius	Unknown	19	Unknown	Philips	0,40	304	344	894
Image 21	TRAIN	Left hand	Unknown	70	Unknown	Philips	0,28	512	509	508
Image 22	TRAIN	Feet	2020	50	M	Siemens	0,54	512	512	358
Image 23	TRAIN	Hands and elbows	2021	16	F	Siemens	0,30	512	1231	1239
Image 24	TRAIN	Shoulder	2020	28	F	Siemens	0,20	512	565	768
Image 25	TRAIN	Left hand	2021	61	F	Siemens	0,45	512	512	410
Image 26	TRAIN	Right hand	2022	62	F	Siemens	0,29	512	512	514
Image 27	TRAIN	Left hand	2021	44	M	Siemens	0,24	768	768	605
Image 28	TRAIN	Left thorax	Unknown	23	M	Siemens	0,38	512	512	991
Image 29	TRAIN	Right thorax	2022	23	M	Siemens	0,38	512	512	991
Image 30	TRAIN	Pelvis	Unknown	2	F	Siemens	0,53	327	454	423
Image 31	TRAIN	Pelvis	Unknown	4	M	Philips	0,33	349	634	565
Image 32	TRAIN	Spine	1990	71	Unknown	GE	0,82	512	512	459
Image 33	TRAIN	Left foot	2021	67	F	Siemens	0,21	554	512	800
Image 34	TRAIN	Spine sever scoliosis (upper part)	Unknown	11	F	Philips	0,49	512	512	344
Image 35	TRAIN	Pediatric scoliosis	Unknown	6	M	Siemens	0,53	316	512	521
Image 36	TRAIN	Severe scoliosis	Unknown	14	F	Toshiba	0,50	512	512	919
Image 37	TRAIN	Standard skull, older subject	Unknown	92	F	Siemens	0,43	511	512	346
Image 38	TRAIN	Spine	Unknown	62	F	Philips	0,40	512	512	661
Image 39	TRAIN	Boths full legs with knees	2003	Unknown	F	GE	0,98	213	282	527
Image 40	TRAIN	Right Thorax	2022	58	M	Siemens	0,36	512	512	1137

nial structures

Image	Usage	Description	AcquisitionDate	Age (year)	Sex	Scanner	Resolution (mm)	SizeX	SizeY	SizeZ
Image 1	TRAIN	Cranioplasty	2018	22	F	Philips	0,43	270	511	235
Image 2	TRAIN	Suspected hemorage	2018	63	M	Philips	0,43	291	512	231
Image 3	TRAIN	Suspected hemorage	2018	53	M	Philips	0,43	286	512	249
Image 4	TRAIN	Cranioplasty	Unknown	48	F	GE	0,52	254	355	209
Image 5	TRAIN	Suspected hemorage	2018	54	F	Philips	0,43	303	487	217
Image 6	TRAIN	Cranioplasty	Unknown	28	F	Siemens	0,43	209	315	191
Image 7	TRAIN	Orbita floor fracture	2021	71	F	Siemens	0,39	271	383	253
Image 8	TRAIN	Suspected hemorage	2018	63	F	Philips	0,43	322	427	227
Image 9	TRAIN	Cranioplasty	2020	47	F	Philips	0,43	318	321	239
Image 10	TRAIN	Intracranial blood vessels	Unknown	51	M	Philips	0,39	274	512	265
Image 11	TRAIN	Suspected intracranial aneurysm	Unknown	65	F	Siemens	0,40	288	384	225

Oculo-crar

Image 12	TRAIN	Tumor	Unknown	69	F	Siemens	0,48	268	329	207
Image 13	TRAIN	Cranioplasty	Unknown	27	M	Philips	0,43	261	512	214
Image 14	TRAIN	Cranioplasty	Unknown	83	M	Toshiba	0,43	344	345	253
Image 15	TRAIN	Cranioplasty	Unknown	60	F	Siemens	0,43	259	512	250
Image 16	TRAIN	Cranioplasty	2019	41	M	Philips	0,45	224	392	214
Image 17	TRAIN	Cranioplasty	2022	56	F	Siemens	0,45	289	312	251
Image 18	TRAIN	Cranioplasty	Unknown	11	M	Philips	0,43	288	358	252
Image 19	TRAIN	Fibrotic dysplacia	Unknown	29	F	Siemens	0,43	254	324	245
Image 20	TRAIN	Normal anatomy	2018	94	M	Philips	0,43	357	512	223
Image 21	TRAIN	Normal anatomy	2018	21	M	Philips	0,43	290	512	212

Mandibulo-cranial structures

Image	Usage	Description	AcquisitionDate	Age (year)	Sex	Scanner	Resolution (mm)	SizeX	SizeY	SizeZ
Image 1	TRAIN	Normal anatomy, two extracted wisdom teeth. No teeth gap	2023	35	F	GE	0,31	512	512	516
Image 2	TRAIN	Intracranial aneurysm (outside image volume)	Unknown	57	F	Siemens	0,43	369	379	333
Image 3	TRAIN	Strong metal Artefacts	Unknown	26	M	GE	0,47	512	512	171
Image 4	TRAIN	small artefacts around the teeth	2020	43	M	Siemens	0,38	512	512	485
Image 5	TRAIN	No teeth but intubated	Unknown	48	F	GE	0,52	290	262	272
Image 6	TRAIN	Orbita floor fracture, no teeth	2021	82	F	Siemens	0,54	279	349	250
Image 7	TRAIN	Orbita floor fracture	2021	71	F	Siemens	0,39	392	476	408
Image 8	TRAIN	Orbita floor fracture	2021	51	F	Siemens	0,56	217	263	307
Image 9	TRAIN	Good quality image with artefacts in the mouth	2018	39	F	GE	0,98	233	190	180
Image 10	TRAIN	Severe head trauma including orbital region and many missing teath in maxilla	2020	52	M	Philips	0,78	512	512	250
Image 11	TRAIN	Normal	Unknown	52	M	Philips	0,39	334	343	312
Image 12	TRAIN	Endast tre tänder i mandibeln	Unknown	66	F	Siemens	0,40	307	348	297
Image 13	TRAIN	Deformed mandibular junction bilateral	Unknown	27	F	Philips	0,43	258	311	249
Image 14	TRAIN	Severe mandible trauma with multiple metal plates	2023	29	M	Philips	0,43	512	512	478
Image 15	TRAIN	Broken mandible, multiple metal plates, still good quality	Unknown	45	F	Siemens	0,34	364	448	401
Image 16	TRAIN	Head trauma, orbital region	2020	55	M	Siemens	0,36	306	399	318
Image 17	TRAIN	Large skull defect, need of cranioplasty	2023	23	M	GE	0,49	489	395	503
Image 18	TRAIN	Madible cut and cranial trauma + small screws	2023	33	M	Siemens	0,41	457	392	409
Image 19	TRAIN	Candidate for ortognatic surgery, metal braces	2020	47	F	Philips	0,43	278	301	261

genital heart defects

Image	Usage	Description	AcquisitionDate	Age (year)	Sex	Scanner	Resolution (mm)	SizeX	SizeY	SizeZ
Image 1	TRAIN		Unknown	Unknown	Unknown	Siemens	0,59	271	279	440
Image 2	TRAIN		2020	0,33	M	Siemens	0,25	382	512	325
Image 3	TRAIN		Unknown	Unknown	Unknown	Siemens	0,28	512	512	371
Image 4	TRAIN		2019	Unknown	M	Siemens	0,26	512	512	417
Image 5	TRAIN		2020	Unknown	F	Siemens	0,30	378	454	289
Image 6	TRAIN		2021	0,02	M	Siemens	0,26	512	512	299
Image 7	TRAIN		Unknown	Unknown	Unknown	Siemens	0,35	512	512	213
Image 8	TRAIN		Unknown	Unknown	Unknown	Siemens	0,57	512	512	268
Image 9	TRAIN		2021	1	M	Siemens	0,30	512	512	411
Image 10	TRAIN		2021	0,25	M	Siemens	0,30	512	512	377
Image 11	TRAIN		2021	1	M	Siemens	0,31	512	512	376
Image 12	TRAIN		2021	2	M	Siemens	0,35	512	512	402
Image 13	TRAIN		2021	8	M	Siemens	0,41	512	512	428
Image 14	TRAIN		2021	1	M	Siemens	0,39	512	512	355
Image 15	TRAIN		2021	3	M	Siemens	0,39	512	512	346
Image 16	TRAIN		2021	17	M	Siemens	0,48	512	512	435
Image 17	TRAIN		2021	2	F	Siemens	0,35	512	512	499
Image 18	TRAIN		2021	2	F	Siemens	0,35	512	512	470
Image 19	TRAIN		2021	1	M	Siemens	0,30	512	512	411
Image 20	TRAIN		2021	0,25	F	Siemens	0,26	512	512	257

Con

Image 21	TRAIN		2021	0,17	M	Siemens	0,27	512	512	410
Image 22	TRAIN		2021	3	F	Siemens	0,37	512	512	395
Image 23	TRAIN		2021	0,17	F	Siemens	0,29	512	512	414
Image 24	TRAIN		2021	0,33	F	Siemens	0,30	512	512	471
Image 25	TRAIN		2021	0,50	F	Siemens	0,34	512	512	452
Image 26	TRAIN		2021	0,25	M	Siemens	0,28	512	512	371
Image 27	TRAIN		2021	0,25	F	Siemens	0,26	512	512	238
Image 28	TRAIN		2021	0,33	F	Siemens	0,30	512	512	410
Image 29	TRAIN		2020	0,33	F	Siemens	0,30	512	512	391
Image 30	TRAIN		2021	14	F	Siemens	0,57	512	512	386
Image 31	TRAIN		2019	Unknown	M	Siemens	0,41	252	306	350
Image 32	TRAIN		2019	3	M	Siemens	0,30	512	512	333

Fetal anatomy

Image	Usage	Description	AcquisitionDate	Age (year)	Gestational age (	Sex	Scanner	Resolution (mm)	SizeX	SizeY	SizeZ
Image 1	TRAIN		2020	Unknown	34,71	F	Siemens	1,56	256	168	184
Image 2	TRAIN		2016	Unknown	35,86	F	Siemens	0,82	416	512	292
Image 3	TRAIN		2020	Unknown	34,57	F	Siemens	0,96	416	380	216
Image 4	TRAIN		2017	Unknown	36,71	F	Siemens	0,78	512	336	308
Image 5	TRAIN		2016	Unknown	36,00	F	Siemens	0,78	512	336	308
Image 6	TRAIN		2016	Unknown	35,57	F	Siemens	0,78	512	336	307
Image 7	TRAIN		2020	Unknown	33,57	F	Siemens	0,78	512	464	307
Image 8	TRAIN		2020	Unknown	32,29	F	Siemens	1,41	256	256	205
Image 9	TRAIN		2016	Unknown	36,57	F	Siemens	0,78	336	512	307
Image 10	TRAIN		2017	Unknown	33,71	F	Siemens	0,78	512	336	308
Image 11	TRAIN		2017	Unknown	39,29	F	Siemens	1,56	256	168	120
Image 12	TRAIN		2020	Unknown	33,43	F	Siemens	1,64	208	256	146
Image 13	TRAIN		2017	Unknown	38,14	F	Siemens	0,82	512	352	293
Image 14	TRAIN		2015	Unknown	38,29	F	Siemens	2,00	190	208	120
Image 15	TRAIN		2020	Unknown	33,14	F	Siemens	1,56	256	168	184
Image 16	TRAIN		2018	Unknown	35,86	F	Siemens	0,78	512	416	246
Image 17	TRAIN		2016	Unknown	35,00	F	Siemens	0,78	512	336	308
Image 18	TRAIN		2015	Unknown	36,57	F	Siemens	0,96	380	416	216
Image 19	TRAIN		2016	Unknown	38,29	F	Siemens	0,78	512	336	308
Image 20	TRAIN		2016	Unknown	29,86	F	Siemens	0,78	512	336	307

Pulmonary-tracheal structures

Image	Usage	Description	AcquisitionDate	Age (year)	Sex	Scanner	Resolution (mm)	SizeX	SizeY	SizeZ
Image 1	TRAIN	Intracranial aneurysm (with contrast)	2021	57	F	Siemens	0,43	503	512	491
Image 2	TRAIN	Lung cancer, no contrast on board. Steroid treated	1994	42	F	Siemens	0,65	512	512	270
Image 3	TRAIN	Esophagus atresia, brachialis compression of trachea (contrast)	2023	0,3	M	Siemens	0,35	249	365	375
Image 4	TRAIN	Anonymous repaired aortic arch (contrast)	Unknown	8	M	Siemens	0,70	512	512	502
Image 5	TRAIN	Congenital heart disease with calcified graft, metal artefacts (contrast)	2020	12	M	Siemens	0,57	239	306	386
Image 6	TRAIN	GUCH with atrial fibrillation (contrast)	2020	30	F	Siemens	0,60	512	512	386
Image 7	TRAIN	GUCH, anominal coronary vessels, APC (contrast)	2020	35	M	Siemens	0,35	512	512	600
Image 8	TRAIN	Congenital heart disease, TGA (contrast)	2021	3	M	Siemens	0,39	353	512	329
Image 9	TRAIN	Normal subject from heart failure cohort (no contrast)	2015	71	F	Siemens	0,78	512	512	220
Image 10	TRAIN	Repaired congenital heart disease, calcified VSD patch (contrast)	2019	41	M	Siemens	0,50	455	468	355
Image 11	TRAIN	MAPC, enlarged aortic root (contrast)	2023	24	M	Siemens	0,46	491	717	667
Image 12	TRAIN	Aortic ring (contrast)	2022	0,4	F	Siemens	0,30	512	512	371
Image 13	TRAIN	Lung cancer (no contrast)	1993	78	M	Siemens	0,79	322	396	364
Image 14	TRAIN	Sclerotic spine (no contrast, quite poor image quality)	2018	6	M	Siemens	0,53	316	512	413
Image 15	TRAIN	Uni ventricle, trachea compression from aortic arch (contrast)	2023	0,25	M	Siemens	0,33	315	501	341
